# Supplementary material for: LIM-Homeodomain Transcription Factor LHX4 Is Required for the Differentiation of Retinal Rod Bipolar Cells and OFF-Cone Bipolar Subtypes
Source: Cell Rep. Author manuscript; Available in PMC 2022 Jun 30. (PMC9245082; doi:10.1016/j.celrep.2020.108144)
Supplement: 1 [file NIHMS1629509-supplement-1.pdf]

Cell Reports, Volume *32*

Supplemental Information

LIM-Homeodomain Transcription Factor LHX4 Is Required for the Differentiation of Retinal Rod Bipolar Cells and OFF-Cone Bipolar Subtypes

Xuhui Dong, Hua Yang, Xiangtian Zhou, Xiaoling Xie, Dongliang Yu, Luming Guo, Mei Xu, Wenjun Zhang, Guoqing Liang, and Lin Gan

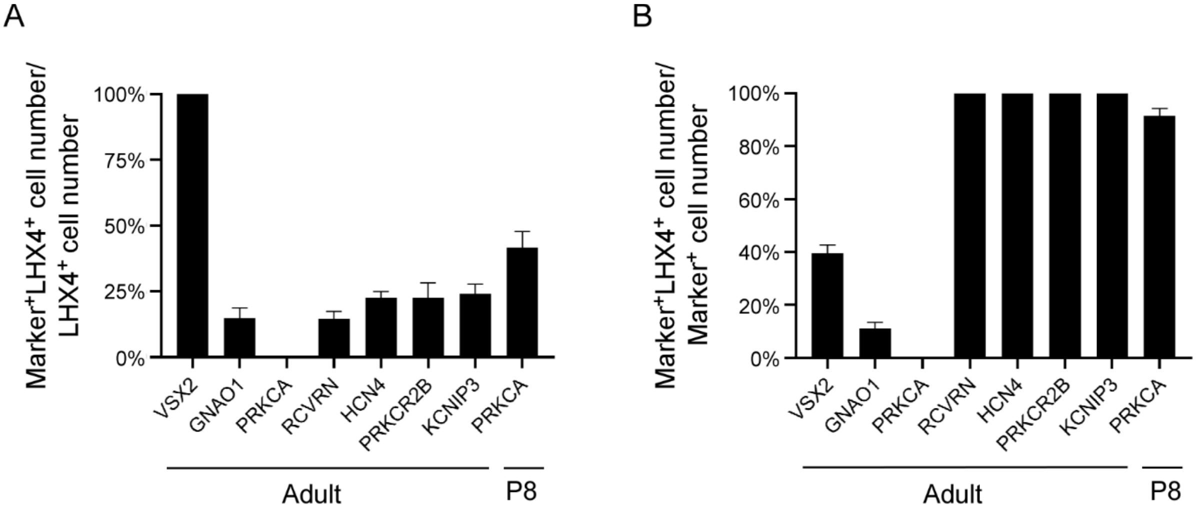

Figure S1. Quantification of LHX4 expression in bipolar subtypes. Related to Figure 1. (A) The percentage of $LHX4^+$ cells expressed different bipolar subtype markers. (B) The percentage of different bipolar subtype markers expressed LHX4.

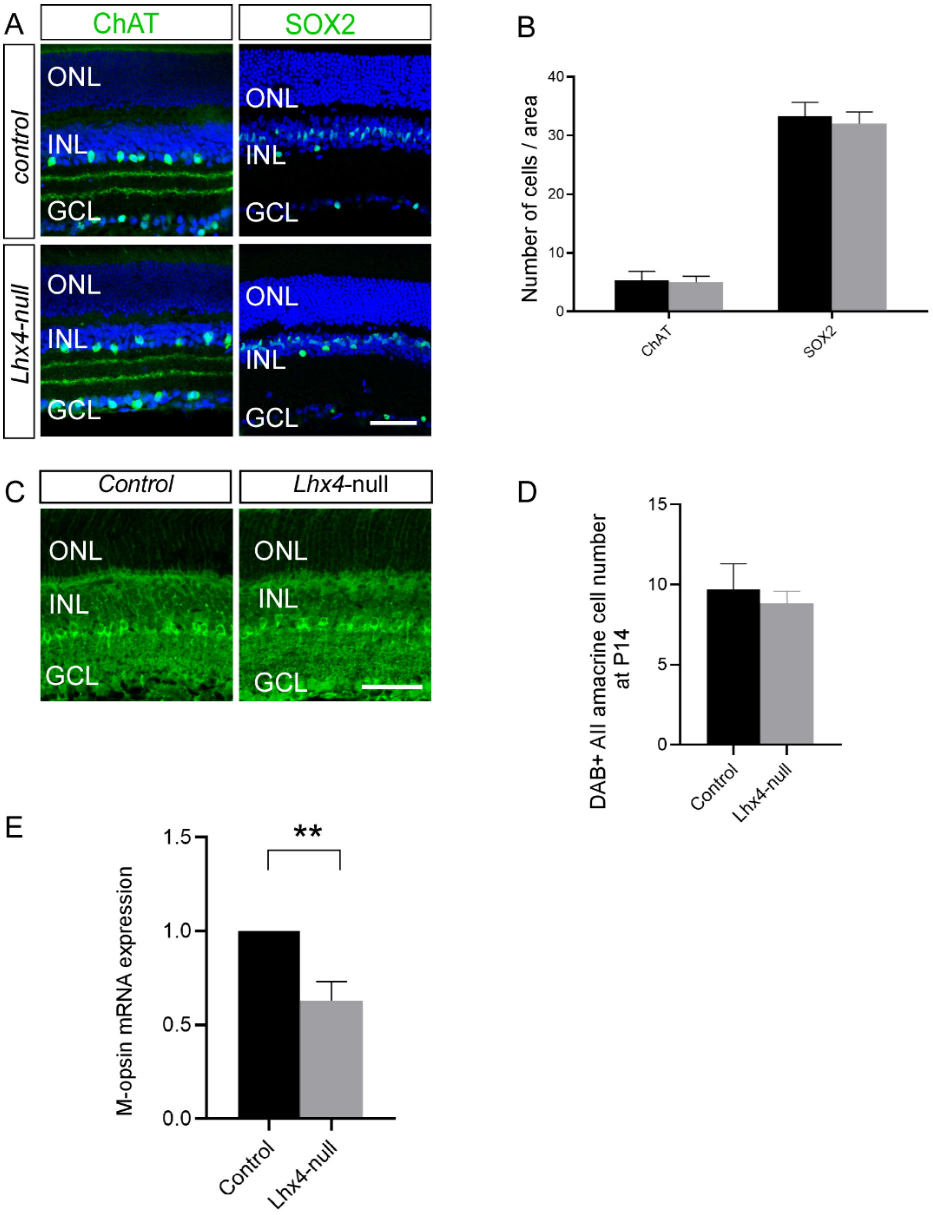

Figure S2. The effect of *Lhx4* inactivation on the generation of Müller glial cells and amacrine cells and the expression of M-opsin. Related to Figure 3. (A) The number of $SOX2^+$ Müller glial cells and $ChAT^+$ starburst ACs in the *Lhx4*-null retina was comparable to that in the control at adult. (B) Quantification of the cell numbers per imaging area ($n \geq 3$). (C) Immunolabeling for DAB1 showed that AII ACs were generated normally in the *Lhx4*-null retina at P14. (D) Quantification of $DAB1^+$ AII amacrine cell number in the *Lhx4*-null retina and control at P14. (E) ddPCR analysis showed *M-opsin* mRNA was significantly reduced by 37.0% in the adult *Lhx4*-null retina. Data are represented as mean ± SD. $^{**}p < 0.01$. Scale bars, 50 μm.

A

Category	Gene
Glutamate receptor signaling pathway	*Grm6*, *Grik1*
Anterograde trans-synaptic signaling	*Gabrr3*, *Grik1*, *Mchr1*
Chemical synaptic transmission	*Gabrr3*, *Grik1*, *Mchr1*
Modulation of chemical synaptic transmission	*Grm6*, *Grik1*
Regulation of synaptic transmission, glutamatergic	*Grm6*, *Grik1*
Adenylate cyclase-inhibiting G-protein coupled receptor signaling pathway	*Grm6*, *Mchr1*
Indolalkylamine metabolic process	*Tph1*, *Aanat*
Cellular biogenic amine biosynthetic process	*Tph1*, *Aanat*
Ion transmembrane transport	*Gabrr3, Atp4a, Ryr3*
Regulation of cell communication	*Grik1, Samsn1*

B

Category	Gene
Glutamate receptor signaling pathway	*Trpm1*, *Grm6*, *Grik1*
Anterograde trans-synaptic signaling	*Gabrr3*; *Glra1*, *Gabrr1*, *Grik1*
Chemical synaptic transmission	*Gabrr3*; *Glra1*, *Gabrr1*, *Grik1, Cdh8*
Gamma-aminobutyric acid signaling pathway	*Gabrr3*; *Gabrr1*
Synaptic transmission, glutamatergic	*Grik1, Cdh8*
G-protein coupled glutamate receptor signaling pathway	*Trpm1, Grm6*
Calcium ion transport	*Trpm1*, *Trpc7*, *Ccr5*, *Ryr3*
Calcium ion transmembrane transport	*Trpm1*, *Trpc7*, *Ryr3*
Response to alcohol	*Glra1*, *Ccr5*
Ion transmembrane transport	*Gabrr3*; *Glra1*, *Gabrr1*, *Ryr3*

Figure S3. RNA-Seq analysis shows that loss of *Lhx4* results in differentially expressed genes associated with synapses. Related to Figure 5. (A) Genes downregulated by loss of *Lhx4* in the top 10 categories of GO enrichment at P6. (B) Genes downregulated by loss of *Lhx4* in the top 10 categories of GO enrichment at P7.

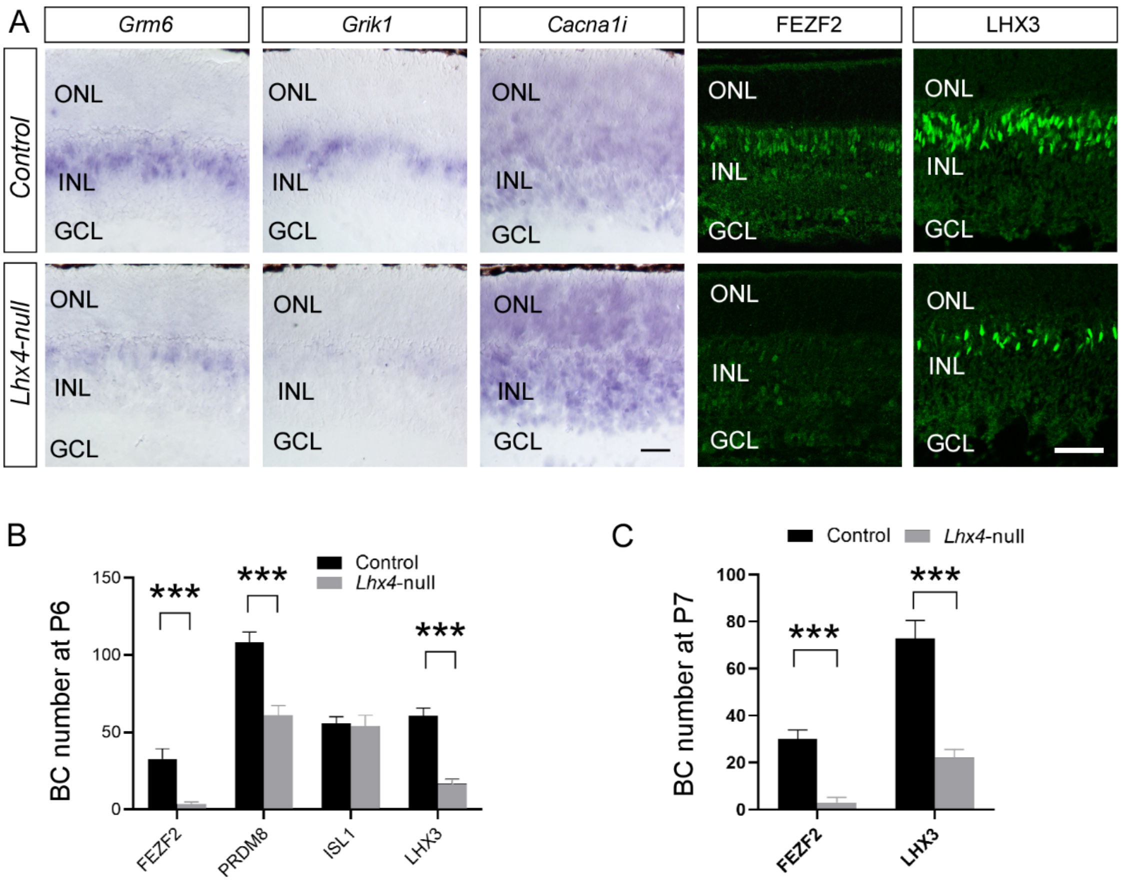

Figure S4. In situ hybridization and immunostaining confirmation of differentially expressed genes. Related to Figure 5. (A) The expression of *Grik1, Grm6*, LHX3, and FEZF2 was significantly reduced in *Lhx4*-null retinas, while the expression of *Cacna1i* was increased in *Lhx4*-null retinas at P7. (B) Quantification of $FEZF2^{+}$, $PRDM8^{+}$, $ISL1^{+}$, and $LHX3^{+}$ BCs in the *Lhx4*-null retina and control at P6. (C) Quantification of $FEZF2^{+}$ and $LHX3^{+}$ BCs in the *Lhx4*-null retina and control at P7. Data are represented as mean ± SD. ***$p < 0.001$. Scale bars, 50 μm.

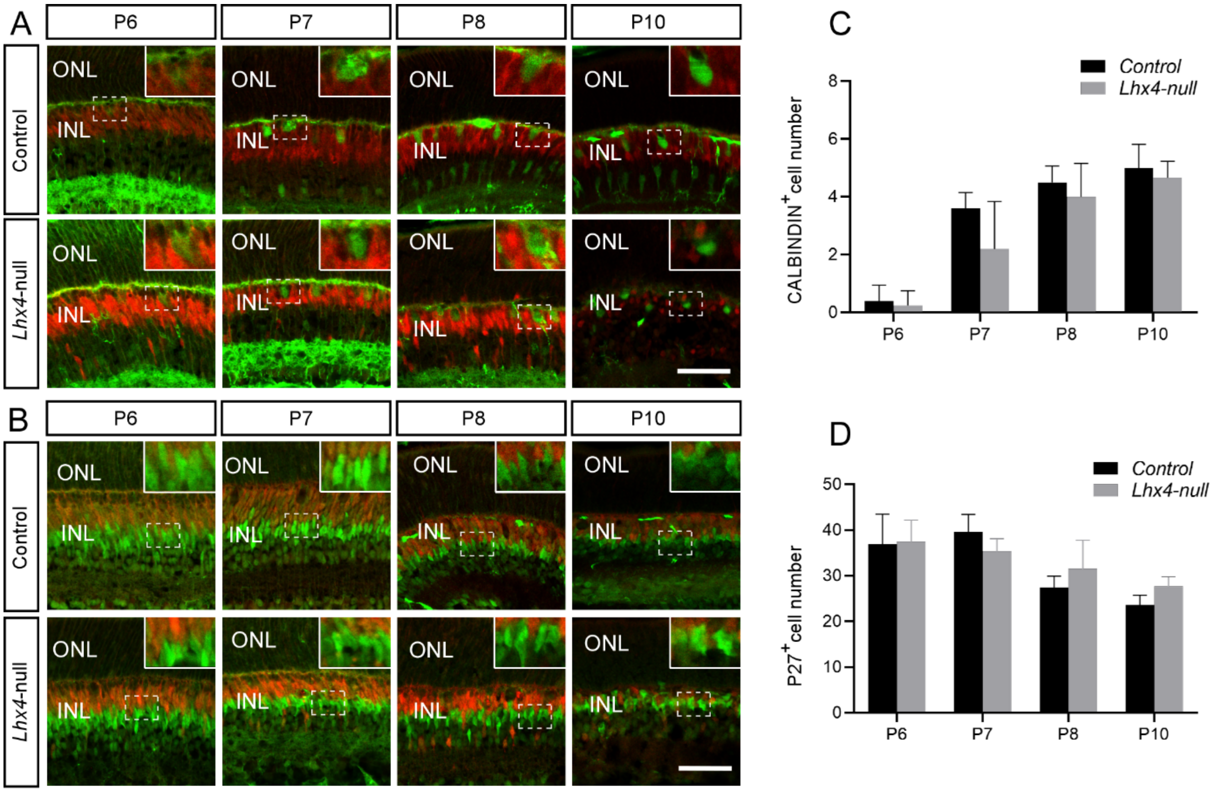

Figure S5. The expression of tdTomato is not detected in the horizontal cells and Müller glia cells in the *Lhx4*-null retina. Related to Figure 6. (A) CALBINDIN$^+$ horizontal cells did not express tdTomato in the control or the *Lhx4*-null retina at P6, P7, P8 and P10. (B) No tdTomato expression was detected in Müller glia cells (p27kip1$^+$) of the control and the *Lhx4*-null retina at P6, P7, P8 and P10. (C) Quantification of CALBINDIN$^+$ horizontal cell number in the *Lhx4*-null retina and control. (D) Quantification of P27$^+$ Müller glia cell number in the *Lhx4*-null retina and control at P6, P7, P8 and P10. Scale bars, 50 µm.
