# Supplementary material for: LIM-Homeodomain Transcription Factor LHX4 Is Required for the Differentiation of Retinal Rod Bipolar Cells and OFF-Cone Bipolar Subtypes
Source: Cell Rep. Author manuscript; Available in PMC 2022 Jun 30. (PMC9245082; doi:10.1016/j.celrep.2020.108144)
Supplement: 4 [file NIHMS1629509-supplement-4.pdf]

Cell Reports

Article

LIM-Homeodomain Transcription Factor LHX4 Is Required for the Differentiation of Retinal Rod Bipolar Cells and OFF-Cone Bipolar Subtypes

Graphical Abstract

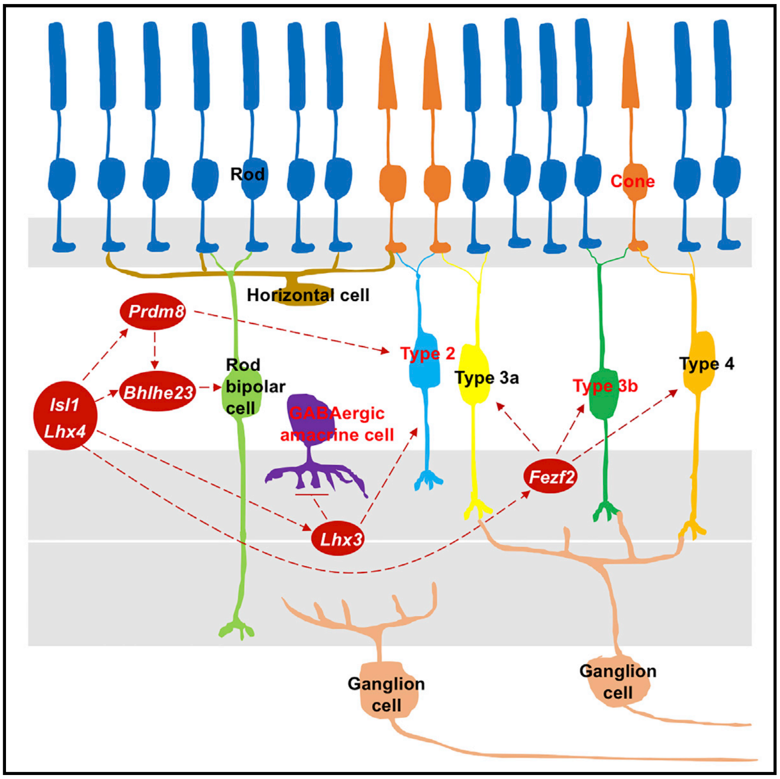

Highlights

- *Lhx4* null mice display a visual defect resembling human CSNB
- Loss of *Lhx4* results in the loss of rod bipolar cells (RBCs) and rod-connecting BCs
- *Lhx4* regulates cell fate choice of type 2 BCs over GABAergic amacrine cells through *Lhx3*
- Overexpression of *Bhlhe23* partially rescues RBC development in the absence of *Lhx4*

Authors

Xuhui Dong, Hua Yang, Xiangtian Zhou, ..., Wenjun Zhang, Guoqing Liang, Lin Gan

Correspondence

lianggq@hznu.edu.cn (G.L.), ligan@augusta.edu (L.G.)

In Brief

Dong et al. show that the loss of *Lhx4* in mice results in the loss of rod bipolar cells and rod-connecting bipolar cells and in a visual defect resembling human congenital stationary night blindness. *Lhx4*, together with *Isl1*, acts upstream of *Bhlhe23*, *Prdm8*, *Fezf2*, and *Lhx3* to regulate bipolar cell development.

Dong et al., 2020, Cell Reports *32*, 108144
September 15, 2020 © 2020 The Author(s).
https://doi.org/10.1016/j.celrep.2020.108144

CellPress

Cell Reports

CellPress
OPEN ACCESS

Article

LIM-Homeodomain Transcription Factor LHX4 Is Required for the Differentiation of Retinal Rod Bipolar Cells and OFF-Cone Bipolar Subtypes

Xuhui Dong,[1,2,3,9] Hua Yang,[3,4,9] Xiangtian Zhou,[5] Xiaoling Xie,[2,3] Dongliang Yu,[6] Luming Guo,[1,3] Mei Xu,[1,3,7] Wenjun Zhang,[3,8] Guoqing Liang,[7,*] and Lin Gan[2,3,10,*]
[1]College of Life Sciences, Zhejiang University, Hangzhou, Zhejiang 310058, China
[2]Department of Neuroscience & Regenerative Medicine, Medical College of Georgia, Augusta University, Augusta, GA 30912, USA
[3]Department of Ophthalmology and Flaum Eye Institute, University of Rochester, Rochester, NY 14642, USA
[4]Department of Otolaryngology, Peking Union Medical College Hospital, Chinese Academy of Medical Sciences and Peking Union Medical College, Beijing, China
[5]School of Ophthalmology and Optometry, Wenzhou Medical University, Wenzhou, Zhejiang 325027, China
[6]College of Life Science and Medicine, Zhejiang Sci-Tech University, Hangzhou 310018, China
[7]Institute of Life Sciences, Hangzhou Normal University, Hangzhou, Zhejiang 310036, China
[8]Department of Plastic Surgery, Changzheng Hospital, Shanghai 20003, China
[9]These authors contributed equally
[10]Lead Contact
*Correspondence: lianggq@hznu.edu.cn (G.L.), ligan@augusta.edu (L.G.)
https://doi.org/10.1016/j.celrep.2020.108144

SUMMARY

Retinal bipolar cells (BCs) connect with photoreceptors and relay visual information to retinal ganglion cells (RGCs). Retina-specific deletion of *Lhx4* in mice results in a visual defect resembling human congenital stationary night blindness. This visual dysfunction results from the absence of rod bipolar cells (RBCs) and the loss of selective rod-connecting cone bipolar cell (CBC) subtypes and AII amacrine cells (ACs). Inactivation of *Lhx4* causes the apoptosis of BCs and cell fate switch from some BCs to ACs, whereas *Lhx4* overexpression promotes BC genesis. Moreover, *Lhx4* positively regulates *Lhx3* expression to drive the fate choice of type 2 BCs over the GABAergic ACs. *Lhx4* inactivation ablates *Bhlhe23* expression, whereas overexpression of *Bhlhe23* partially rescues RBC development in the absence of *Lhx4*. Thus, by acting upstream of *Bhlhe23*, *Prdm8*, *Fezf2*, *Lhx3*, and other BC genes, *Lhx4*, together with *Isl1*, could play essential roles in regulating the subtype-specific development of RBCs and CBCs.

INTRODUCTION

In the retina, bipolar cells (BCs) are positioned in the inner nuclear layer (INL) between photoreceptors at the outer nuclear layer (ONL) and retinal ganglion cells (RGCs) at the ganglion cell layer (GCL). BCs play the essential role of transmitting visual signals from photoreceptors to RGCs. Previous studies have shown that the specification of BCs depends on the coordinated action of several transcription factor (TF) genes (Burmeister et al., 1996; Hatakeyama et al., 2001; Livne-Bar et al., 2006; Tomita et al., 2000). The loss of homeobox TF gene *Vsx2* results in reduced proliferation of retinal progenitor cells and the absence of BCs in mice (Burmeister et al., 1996). Conversely, overexpression of *Vsx2* in mouse retinas leads to an increase of BCs at the expense of rod photoreceptors (Livne-Bar et al., 2006). In mice null for the basic helix-loop-helix (bHLH) TF genes *Ascl1* and *Neurod4*, BCs are absent and Müller glial cells are significantly increased (Hatakeyama et al., 2001; Tomita et al., 2000). Overexpression of either *Ascl1* or *Neurod4* together with *Vsx2* promotes BC generation at the expense of Müller glial cells, suggesting that these two classes of TFs together specify the bipolar cell fate (Hatakeyama et al., 2001). Among the other TFs essential for BC subtype differentiation, *Fezf2* is required for the differentiation of OFF-cone bipolar cells (CBCs) and functional maturation of ON-CBCs (Suzuki-Kerr et al., 2018). *Bhlhe23*, *Isl1*, and *Prdm8* are required for rod bipolar cell (RBC) differentiation. Mice null for *Bhlhe23*, *Isl1*, or *Prdm8* lose all or most of RBCs and have a defective b-wave in electroretinogram (ERG), resembling human congenital stationary night blindness (CSNB) (Bramblett et al., 2004; Elshatory et al., 2007; Jung et al., 2015).

Human CSNB is a group of retinal disorders that are characterized by impaired night vision. CSNB is classified into two groups based on ERG findings: the Schubert-Bornschein type is characterized by an ERG in which the b-wave is smaller than the a-wave, whereas the Riggs type has proportionally reduced a- and b-waves. In addition, the Schubert-Bornschein CSNB with BC dysfunction is further divided into complete and incomplete CSNB (cCSNB and icCSNB, respectively) (Zeitz et al., 2015). Genetic studies have identified several genes associated with cCSNB, namely, *NYX*, *GRM6*, *GPR179*, and *TRPM1*, or linked to

This is an open access article under the CC BY license (http://creativecommons.org/licenses/by/4.0/).

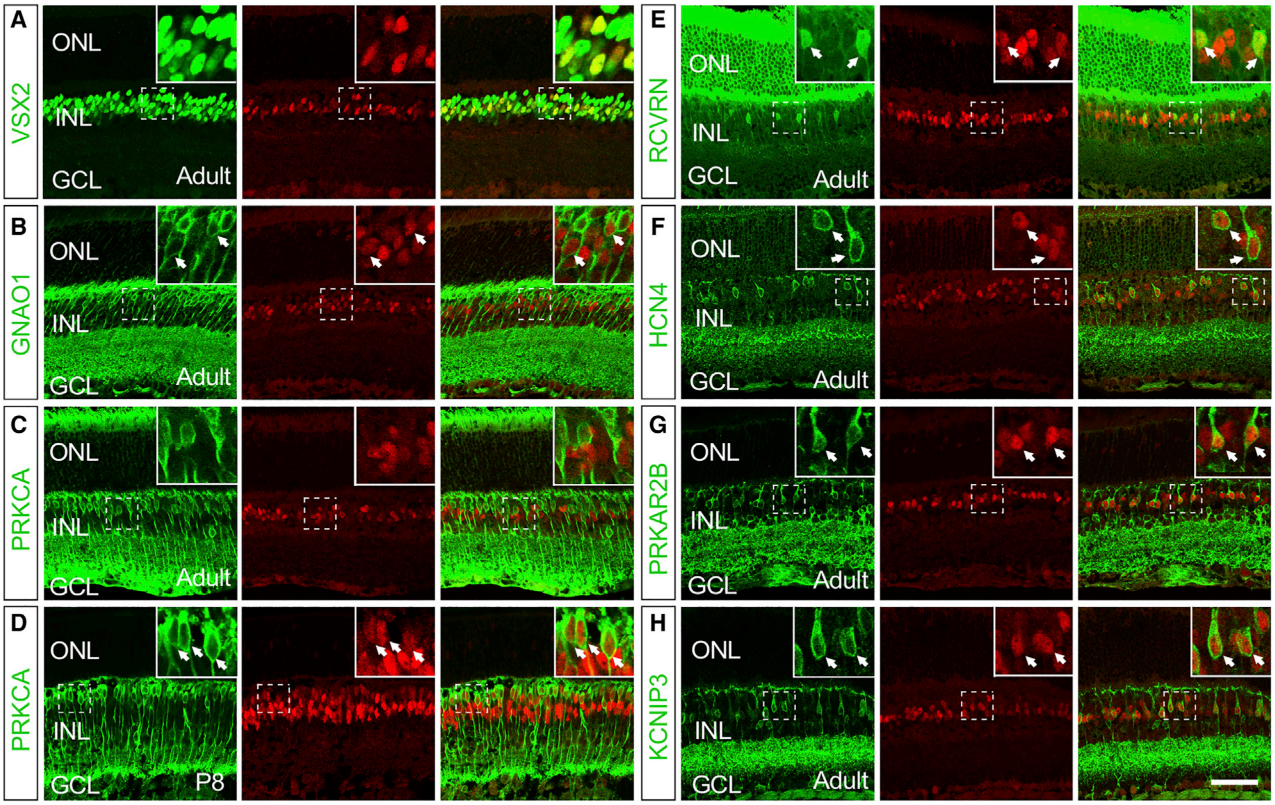

Figure 1. LHX4 Is Expressed in BC Subtypes

Confocal images showing LHX4 antibody (red) co-labeling with BC subtype markers (green) in wild-type retinal sections. Arrows indicate cells with co-expression of LHX4 and BC subtype markers.

(A) All LHX4-expressing cells were VSX2$^+$ BCs.

(B) LHX4 expression partially overlapped that of GNAO1 in the INL.

(C) In the adult retina, LHX4 was not expressed in the PRKCA$^+$ RBCs.

(D) Almost all PRKCA$^+$ developing RBCs expressed LHX4 at P8.

(E) RCVRN expression was co-localized with LHX4 in the adult retina.

(F) All HCN4$^+$ BCs expressed LHX4 in the adult retina.

(G) PRKAR2B was expressed in LHX4$^+$ BCs in the adult retina.

(H) All KCNIP3$^+$ BCs were LHX4$^+$ in the adult retina. Scale bar, 50 μm.

See also Figure S1.

icCSNB, namely, *CACNA1F*, *CABP4*, and *CACNA2D4* (Zeitz et al., 2015). However, the molecular mechanisms underlying this group of genetically heterogeneous diseases are not fully understood.

LIM-homeodomain (HD) TF LHX4 is expressed in the retina, pituitary gland, and spinal cord of developing and adult mice (Blackshaw et al., 2004; Liu et al., 2002; Sharma et al., 1998; Sheng et al., 1997). Evidence of LHX4 expression in retinal BCs argues for its role in the development and function of BCs. In this study, by using a conditional knockout approach to inactivate *Lhx4* in developing mouse retinas, we demonstrated that *Lhx4* acts upstream of *Bhlhe23*, *Prdm8*, *Fezf2*, *Lhx3*, and other BC genes in the differentiation pathway of BCs and that *Lhx4* is essential for the differentiation of RBCs and CBC subtypes and the inhibition of GABAergic amacrine cells (ACs). In *Lhx4* null retinas, RBCs are missing and rod-connecting CBCs are nearly absent, resulting in the absence of scotopic ERG b-wave. These findings reveal a role of *Lhx4* in the retina and implicate *LHX4* as a candidate gene for human CSNB.

RESULTS

LHX4 Expression in BC Subtypes

Previously, we have shown that LHX4 is expressed in a selective group of developing and adult retinal BCs (Balasubramanian et al., 2014; Elshatory et al., 2007). In order to define the BC subtypes expressing LHX4, we co-immunolabeled sections of adult mouse retinas with anti-LHX4 and various BC markers. Co-labeling of LHX4 and VSX2, a pan-BC marker (Jung et al., 2015), showed that all LHX4$^+$ cells were VSX2$^+$ BCs and 39.8% of VSX2$^+$ BCs were LHX4$^+$ cells (Figures 1A, S1A, and S1B). Among the ON-BC markers, GNAO1 is expressed by all ON-BCs (Bramblett et al., 2004; Haverkamp and Wässle, 2000). Approximately 15.0% of LHX4$^+$ cells expressed GNAO1 and 11.1% of GNAO1$^+$ cells expressed LHX4 (Figure 1B, arrows, S1A, and S1B). LHX4 was not detected in the cells expressing PRKCA (Figures 1C, S1A, and S1B), a marker for RBCs, and a small population of ACs in adult retinas (Haverkamp et al., 2003). Thus, these

LHX4$^+$/GNAO1$^+$ ON-BCs are all ON-CBCs. These results are consistent with previous studies (Balasubramanian et al., 2014; Shekhar et al., 2016). We further analyzed the expression of LHX4 in developing retinas at post-natal day 8 (P8), a stage near the peak of RBC formation and with a high level of LHX4 expression (Elshatory et al., 2007). As shown in Figures 1D, S1A, and S1B, co-immunolabeling results revealed that almost all PRKCA$^+$ RBCs (91.5%) expressed LHX4 and 41.8% of LHX4$^+$ cells expressed PRKCA at P8. Therefore, LHX4 is transiently expressed in RBCs during retinal development, consistent with the single-cell RNA sequencing (scRNA-seq) study (Clark et al., 2019).

To further characterize the subtype-specific expression of LHX4 in CBCs, we co-stained sections of adult retinas with anti-LHX4 and several CBC subtype markers. Recoverin (RCVRN) marks type 2 OFF-CBCs (Feng et al., 2006). All RCVRN+ CBCs expressed LHX4 and represent ~14.4% of LHX4$^+$ BCs (Figures 1E, S1A, and S1B). HCN4 is a marker of type 3a OFF-CBCs (Mataruga et al., 2007). We found that all of the HCN4$^+$ BCs expressed LHX4 and accounted for ~22.3% of LHX4$^+$ cells (Figures 1F, S1A, and S1B). Similarly, co-labeling of LHX4 and PRKAR2B, a marker of type 3b OFF-CBCs (Mataruga et al., 2007), showed that all of the PRKAR2B$^+$ BCs expressed LHX4 and represented ~22.1% of LHX4$^+$ cells (Figures 1G, S1A, and S1B). Calsenilin (KCNIP3) marks type 4 OFF-CBCs (Haverkamp et al., 2008), and all of the KCNIP3$^+$ BCs expressed LHX4, accounting for ~23.9% of LHX4$^+$ cells (Figures 1H, S1A, and S1B). The above results are consistent with the previous single-cell transcriptomics study (Shekhar et al., 2016). Collectively, we have shown that LHX4 is transiently expressed in RBCs during retinal development and is expressed in ON-CBCs and four subtypes of OFF-CBCs in adult retinas.

Severe Retinal Dysfunction in *Lhx4* Null Mice

To investigate the function of *Lhx4* in the retina, we generated *Lhx4*-tdTomato reporter gene knockin (*Lhx4^{tdT}*) and conditional knockout (*Lhx4^{loxP}*) mouse lines (Dong et al., 2019) and used *Six3-Cre* mice to specifically inactivate *Lhx4* in the retina. To assess the effect of *Lhx4* inactivation on retinal function, we performed scotopic and photopic ERG experiments in *Lhx4$^{tdT/loxP}$; Six3-Cre* (*Lhx4* null) and control (wild type or *Lhx4$^{loxP/+}$*) mice. Compared to the scotopic b-wave, which detects a rod response from BCs, of about 344.3 ± 77.0 μV in control mice at 2 months of age, the scotopic b-wave was absent in *Lhx4* null mice (Figures 2A and 2D). In *Lhx4* null mice, the mean b-wave of combined rod-cone response was reduced by 77.7% compared to that of the control (154.8 ± 43.2 μV versus 693.7 ± 122.1 μV, p = 1.023577e-015), and the mean a-wave of combined rod-cone response was reduced by 34.0% compared to that of the control (174.2 ± 74.2 μV versus 264.1 ± 55.7 μV, p = 0.0007) (Figures 2B and 2D). The b-wave primarily represents the response from BCs, whereas the a-wave mostly reflects the response from photoreceptors. In the cone response, the photopic b-wave amplitude decreased by 69.8% in *Lhx4* null mice compared to that of the control (42.2 ± 22.0 μV versus 139.9 ± 45.4 μV, p = 2.256575e-008) (Figures 2C and 2D). The average a-wave of cone response decreased by 42.6% in *Lhx4* null mice compared to that of the control (9.3 ± 6.4 μV versus 16.2 ± 7.5 μV, p = 0.0165) (Figures 2C and 2D). These ERG studies suggest defects in the development or function of RBCs, CBC subtypes, and photoreceptors in *Lhx4* null mice.

Absence of RBCs in the *Lhx4*-Deficient Retina

To identify the molecular and cellular basis of the ERG abnormality in *Lhx4* null mice, we first performed hematoxylin and eosin (H&E) staining of retinal sections. We found that there were 46.6% fewer cells in the INL of *Lhx4* null retinas than in the control, whereas the number of cells in the ONL and GCL was comparable between the control and *Lhx4* null retinas (Figures 2E and 2I).

The loss of scotopic b-wave in the *Lhx4* null mice prompted us to examine RBCs. We performed anti-PRKCA immunolabeling to determine any change in RBCs and found that the PRKCA$^+$ RBCs were absent in *Lhx4* null mice (Figures 2F and 2J). In the retina, the synapses of BCs in the IPL can be revealed by immunolabeling for vGlut1 and SV2B (Ghosh et al., 2004; Wang et al., 2003). These large, globular RBC terminals in the IPL were most visible in the ON sublamina close to the GCL and were prominently labeled with vGlut1 and SV2B in the control retina but disappeared in the *Lhx4* null retina (Figures 2G, 2H, and 2J, arrows). Together, these results confirm that targeted inactivation of *Lhx4* results in the ablation of the RBCs.

Near-Absence of Rod-Connecting CBCs and Significantly Reduced Type 2 CBCs in the *Lhx4* Null Retina

LHX4 is expressed in specific CBC subtypes (Figures 1E–1H), and the cone response of ERG is reduced in *Lhx4* null mice (Figure 2C). Next, we asked whether CBCs were disrupted in a subtype-specific manner in the *Lhx4* null retina using VSX2 and several subtype markers: ISL1, RCVRN, HCN4, PRKAR2B, and KCNIP3 (Feng et al., 2006; Haverkamp et al., 2008; Mataruga et al., 2007). Compared with the control, the total number of BCs (VSX2$^+$) and ISL1$^+$ ON-BCs (RBCs and ON-CBCs) in the *Lhx4* null retina were reduced by 58.2% and 67.0%, respectively (Figures 3A and 3C). We calculated that the reduction of ISL1$^+$ ON-BCs per imaging area examined (~38 per 230 μm^2) is greater than the number of PRKCA$^+$ RBCs lost per imaging area (~29 per 230 μm^2), indicating a loss of ON-CBCs in *Lhx4* null retinas.

Among the four LHX4-expressing OFF-CBCs, the number of RCVRN$^+$ type 2 CBCs was reduced by 58.6% in the *Lhx4* null retina compared to the control (Figures 3A and 3C). The HCN4$^+$ type CBCs were hardly detected in the *Lhx4* null retina, although the expression of HCN4 in ACs remained in the INL of the *Lhx4* null retina (Figures 3A and 3C). Anti-PRKAR2B immunolabeling revealed that PRKAR2B$^+$ type 3b OFF-CBCs were mostly missing, whereas its expression in ACs remained in the INL of the *Lhx4* null retina (Figures 3A and 3C). Similarly, the KCNIP3$^+$ type 4 OFF-CBCs were nearly absent in the *Lhx4* null retina (Figures 3A and 3C). Because type 3a, 3b, and 4 OFF-CBCs, namely rod-connecting CBCs, provide alternative rod pathways by making direct contacts with rod photoreceptors (Euler et al., 2014; Hack et al., 1999; Haverkamp et al., 2008; Mataruga et al., 2007), the loss of these CBC subtypes might partly be responsible for the absence of scotopic ERG b-wave

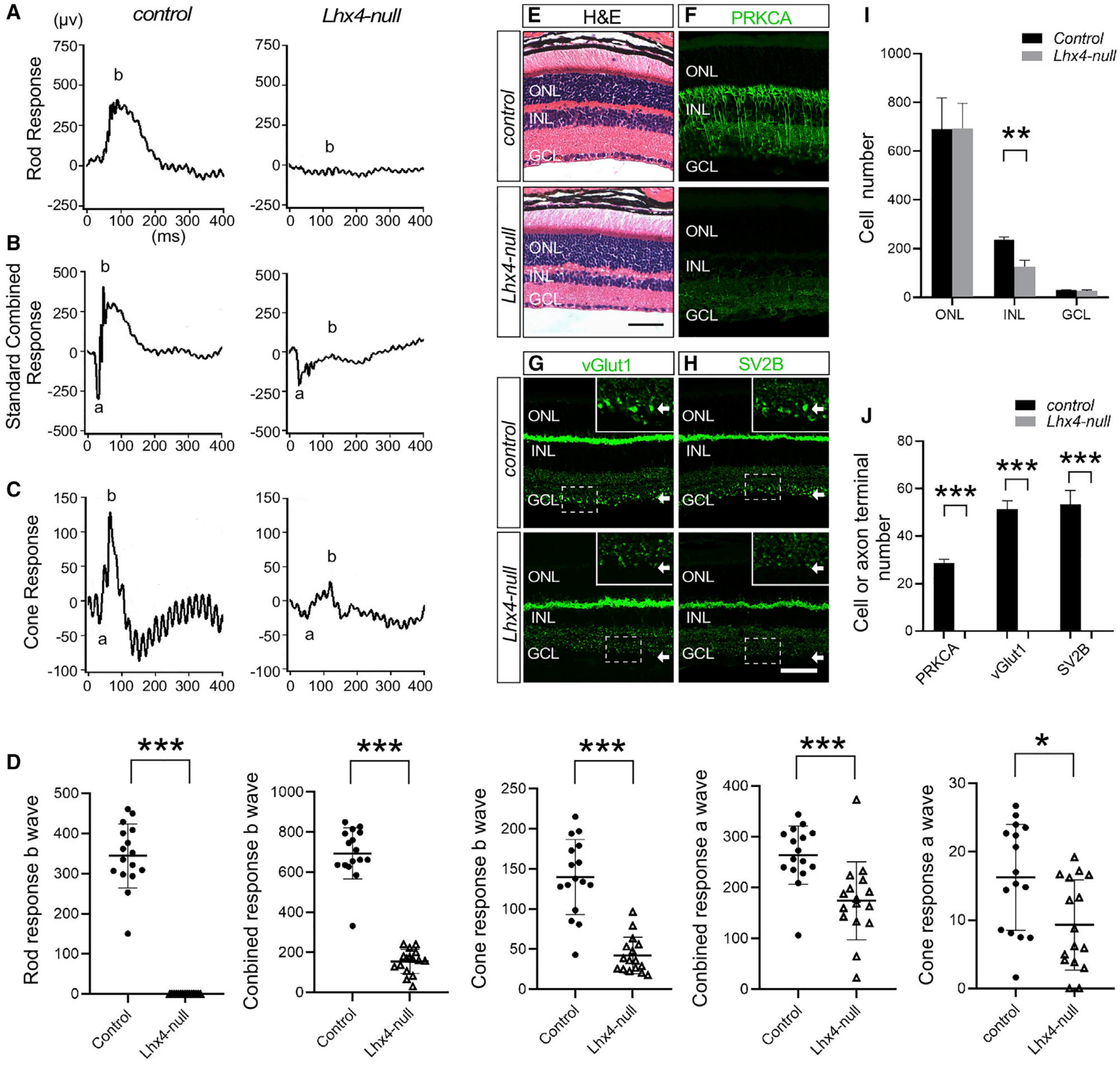

Figure 2. Loss of *Lhx4* Results in the Scotopic ERG b-Wave Defects and the Absence of RBCs

(A) A representative ERG of rod response showed that the scotopic b-wave was absent in *Lhx4* null mice.

(B) A representative ERG of a standard combined response showed that the b-wave was dramatically reduced and a-wave was also significantly decreased in the *Lhx4* null mice compared with the control mice.

(C) A representative cone-ERG response showed that b-wave was reduced and a reduction in a-wave was also detected in the *Lhx4* null mice compared with control mice.

(D) Scatterplots of rod response b-wave, standard combined response b-wave, cone response b-wave, standard combined response a-wave, and cone response a-wave in adult *Lhx4* null and control mice.

(E) Hematoxylin and eosin (H&E) staining revealed that the INL of the adult *Lhx4* null retina was thinner than that of the control.

(F) Anti-PRKCA immunolabeling showed the complete loss of RBCs in the adult *Lhx4* null retina.

(G) The RBC synaptic terminals, which were strongly labeled by anti-vGlut1 in the control, were absent in adult *Lhx4* null retina.

(H) The anti-SV2B-labeled RBC synaptic terminals at the inner edge of the IPL were lost in the adult *Lhx4* null retina.

(I) Quantification of ONL, INL, and GCL cell number in the *Lhx4* null mice and control at adulthood.

(J) Quantification of $PRKCA^+$ RBC number, $vGlut1^+$, and $SV2B^+$ RBC axon terminal number in the *Lhx4* null mice and control at adulthood. *$p < 0.05$; **$p < 0.01$; ***$p < 0.001$. Data are represented as mean ± SD. Scale bar, 50 μm.

CellPress
OPEN ACCESS

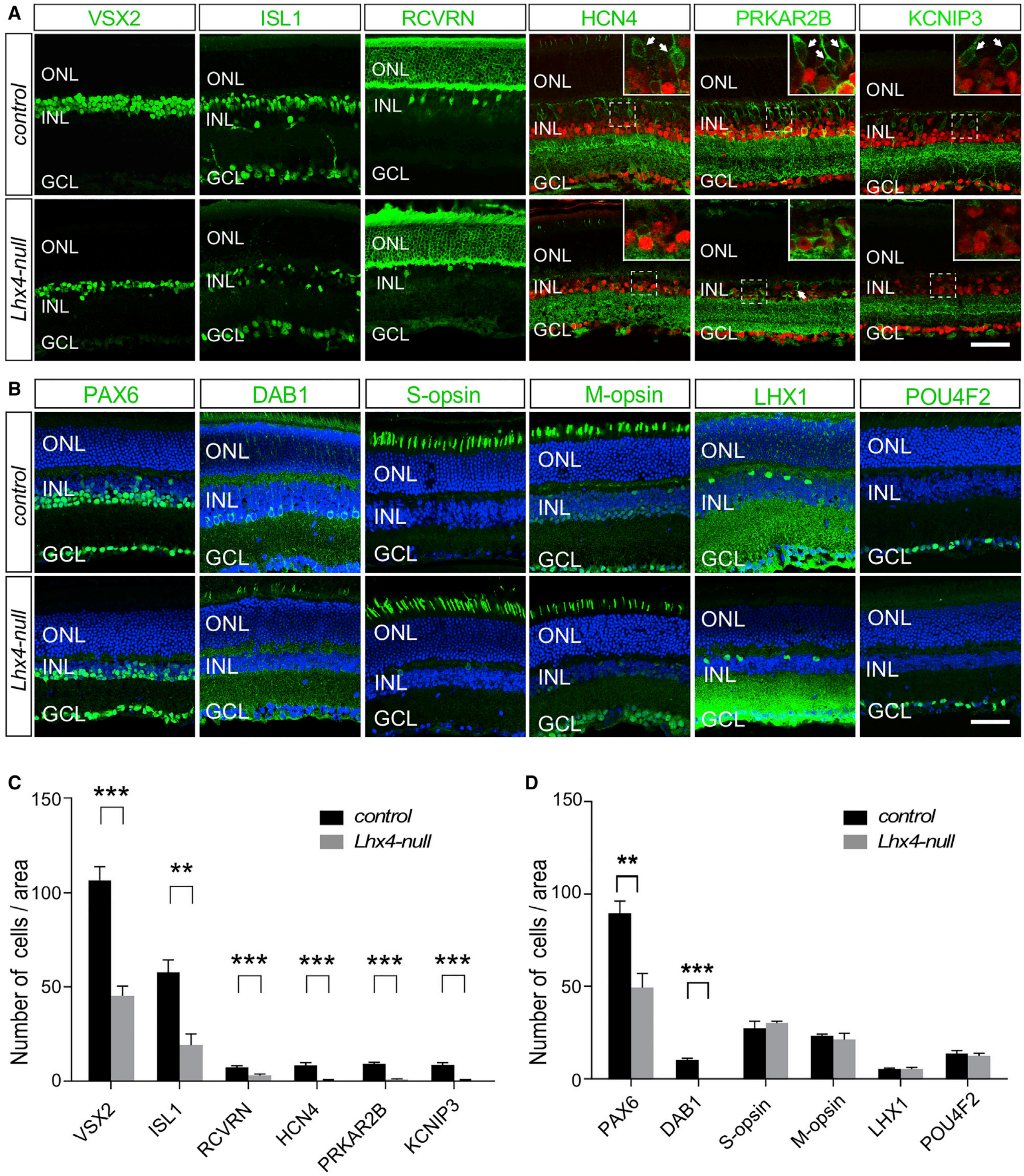

Figure 3. Loss of *Lhx4* Results in the Reduction of Type 2, 3a, 3b, and 4 OFF-CBCs and the Degeneration of All Amacrine Cells

(A) The total number of VSX2$^+$ BCs was greatly reduced in the *Lhx4* null retina compared with the control. The ISL1$^+$ ON-BC population was significantly reduced in the *Lhx4* null retina. The type 2 CBCs labeled by anti-RCVRN were significantly decreased in the *Lhx4* null retina. Anti-PAX6 (red) was co-immunolabeled with BC markers (green) to distinguish BCs (PAX6$^-$) from ACs (PAX6$^+$). HCN4-expressing BCs (HCN4$^+$ PAX6$^-$) were hardly detected in the *Lhx4* null retina. PRKAR2B$^+$ BCs (PRKAR2B$^+$ PAX6$^-$) were greatly diminished in the *Lhx4* null retina. The KCNIP3$^+$ type 4 OFF-CBCs (KCNIP3$^+$ PAX6$^-$) were nearly absent in the *Lhx4* null retina.

(legend continued on next page)

in *Lhx4* null mice. In summary, our results demonstrate that inactivation of *Lhx4* leads to the loss or reduction of ON- and OFF-CBC subtypes.

Loss of AII Amacrine Cells in the Adult *Lhx4* Null Retina

In addition to BCs, the INL contains two other groups of interneurons, amacrine and horizontal cells, as well as Müller glial cells and a small number of displaced RGCs. The number of ACs in the INL was reduced by 45.0% in the adult *Lhx4* null retina (Figures 3B and 3D). The significant loss of BCs of rod pathways in *Lhx4* null retinas propelled us to examine changes in ACs implicated in rod pathways. AII ACs are an essential component of the primary rod pathway and connect RBCs onto the cone bipolar circuitry (Euler et al., 2014; Völgyi et al., 2004). We used anti-DAB1, a marker for AII ACs (Rice and Curran, 2000), to detect the change in AII ACs. Strikingly, DAB1^{+} cells were absent in the adult *Lhx4* null retina (Figures 3B and 3D). In contrast, the number of cholinergic (ChAT^{+}) starburst ACs was unchanged in the *Lhx4* null retina (Figures S2A and S2B). Because *Lhx4* is not expressed in ACs, the reduction of ACs in the adult *Lhx4* null retina, including AII ACs, is likely caused indirectly by the loss of BCs. Thus, we examined whether the generation of AII ACs was affected by the loss of *Lhx4* in the developing retina and found that the DAB1^{+} AII ACs were normally generated in the *Lhx4* null retina at P14 (Figures S2C and S2D).

We also assessed the changes in other neurons of the ONL and GCL. The previous studies show that *Lhx4* is expressed in the cone photoreceptors (Buenaventura et al., 2019; Clark et al., 2019). Immunolabeling analyses of the number of S-cone (S-opsin^{+}) and M-cone (M-opsin^{+}) photoreceptor cells in the *Lhx4* null retina revealed no significant change compared to that of the control (Figures 3B and 3D). However, the expression level of M-opsin in individual cells appeared decreased in the *Lhx4* null retina (Figure 3B), consistent with the Droplet Digital PCR (ddPCR) analysis that the expression of *M-opsin* mRNA was decreased by 37.0% in the *Lhx4* null retina (Figure S2E). The numbers of LHX1^{+} horizontal cells, POU4F2^{+} RGCs, and SOX2^{+} Müller glial cells were normal in the *Lhx4* null retina compared to those of the control (Figures 3B, 3D, S2A, and S2B). Altogether, the loss of *Lhx4* does not affect the number of cone photoreceptors, horizontal cells, Müller glial cells, or RGCs but does result in a reduction of ACs in adults, especially in the absence of AII ACs.

Loss of Nascent BCs through Apoptosis in the *Lhx4* Null Retina

To examine the effect of *Lhx4* null mutation on the development of BCs, we performed H&E staining and anti-VSX2 immunolabeling of retinal sections during the peak of BC differentiation at P6–P10. As shown in Figures 4A and 4B, the H&E staining showed the comparable number of cells in the INL of control and *Lhx4* null retinas at P6. At P7, P8, and P10, the number of cells in the INL of the *Lhx4* null retina was gradually reduced compared with that of the control, whereas no overt change in cell numbers was observed in the ONL and GCL (Figures 4A and 4B). Similarly, anti-VSX2 immunolabeling revealed no significant change in the number of BCs between control and *Lhx4* null retinas at P6 (Figures 4C and 4D), indicating that *Lhx4* is not required for the initial generation of BCs or the onset of VSX2 expression in BCs. At P7, the VSX2^{+} BCs were decreased in *Lhx4* null retinas compared to that of the control, and the loss of VSX2^{+} BCs was also evident in *Lhx4* null retinas at P8 and P10 (Figures 4C and 4D). Thus, although *Lhx4* is dispensable for the initial cell fate specification of pan-BC identity, targeted inactivation of *Lhx4* causes a loss of nascent BCs.

The decrease in BCs at P7 and later stages prompted us to investigate whether the loss of BCs was caused by programmed cell death. Co-immunolabeling with anti-VSX2 and anti-cleaved caspase-3 (CASP3), a marker for apoptosis, revealed about a 2-fold increase of VSX2^{+}/CASP3+ apoptotic cells in the *Lhx4* null retina than that in the control at P7, whereas there was no significant difference at P8 (Figures 4E and 4F). Thus, although *Lhx4* is not required for the generation of BCs, targeted inactivation of *Lhx4* leads to the significant reduction of BCs through apoptosis.

Abnormal Transcriptome of the Developing *Lhx4* Null Retina

To determine the molecular mechanism of *Lhx4* function, we sought to determine the change of transcriptome in the *Lhx4* null retina. RNA-seq was performed with *Lhx4* null and control retinas at P6 and P7. RNA-seq analysis revealed 91 differentially expressed genes (fold change >2; $p < 0.05$) at P6 and 100 differentially expressed genes at P7. Among them, 60 genes were upregulated and 31 genes were downregulated in the *Lhx4* null retina at P6 (Figure 5A; Table S1), whereas 46 genes were upregulated and 54 genes were downregulated in the *Lhx4* null retina at P7 (Figure 5A; Table S2). Gene Ontology (GO) analysis at P6 and P7 revealed that the biological processes related to the function of synapses were highly enriched among the downregulated genes: glutamate receptor signaling pathway, anterograde *trans*-synaptic signaling, chemical synaptic transmission, modulation of synaptic transmission (glutamatergic), and gamma-aminobutyric acid signaling pathway (Figure 5B). The expression of *Grm6*, *Grik1*, *Gabrr3,* and *Mchr1* was significantly reduced in *Lhx4* null retinas at P6 (Figures 5C, 5F, and S3A; Table S1), whereas the expression of *Grm6*, *Grik1*, *Trmp1*, *Gabrr3*, *Glra1*, *Gabrr1*, and *Cdh8* was

(B) Confocal images of adult retinal sections immunolabeled with retinal-cell-specific markers (green) and stained with 4′,6-diamidino-2-phenylindole (DAPI, blue). Immunolabeling with anti-PAX6 showed that the total number of ACs in the INL was significantly reduced in the *Lhx4* null retina compared with the control retina. Anti-DAB1-labeled AII ACs were absent in the *Lhx4* null retina. The number of cone photoreceptors labeled by anti-S-opsin and anti-M-opsin was not significantly changed in the *Lhx4* null retina. However, the expression level of M-opsin in individual photoreceptors appeared reduced in the *Lhx4* null retina. Anti-LHX1 staining revealed no detectable change in horizontal cells between the control and the *Lhx4* null retina. Anti-POU4F2 labeling showed a comparable number of RGCs in the control and the *Lhx4* null retina.

(C) Quantification of the number of different BC subtypes in the control and *Lhx4* null retinas ($n \geq 3$).

(D) Quantification of the cell numbers per imaging area ($n \geq 3$). Data are represented as mean ± SD. **$p < 0.01$; ***$p < 0.001$. Scale bars, 50 μm.

See also Figure S2.

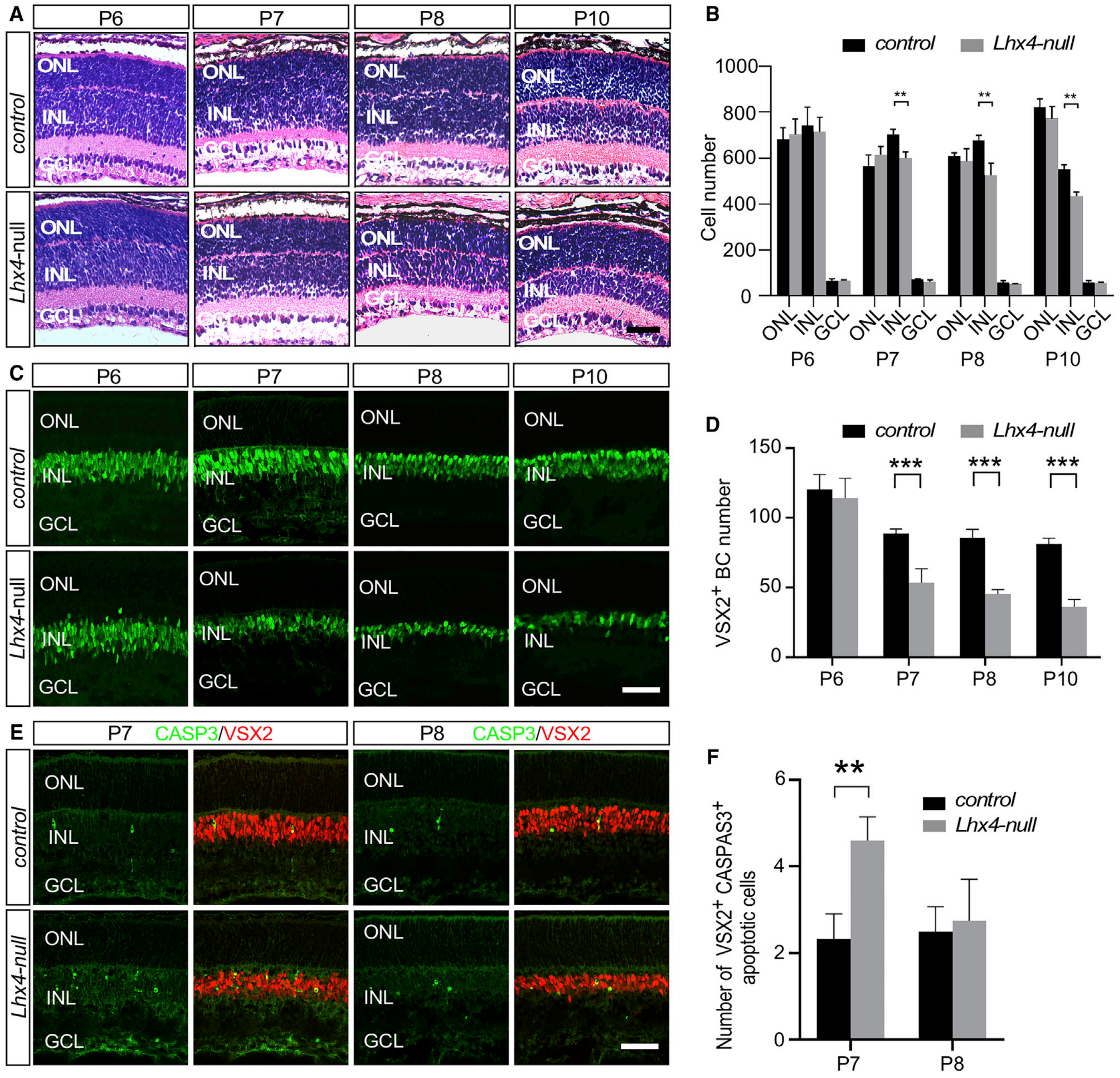

Figure 4. Loss of *Lhx4* Does Not Affect the Initial Generation of BCs but Results in the Apoptosis of Nascent BCs

(A) H&E staining showed that the number of cells in the INL was not significantly changed in the *Lhx4* null retina at P6. At P7–P10, the INL in the *Lhx4* null retinas became thinner than that of the controls.

(B) Quantification of the cells in the ONL, INL, and GCL of the *Lhx4* null and control retinas at P6, P7, P8, and P10.

(C) Anti-VSX2 (green) immunolabeling showed that consistent with H&E staining, the total number of BCs labeled by anti-VSX2 was unchanged in the *Lhx4* null retina at P6 but was reduced in the *Lhx4* null retina at P7–P10.

(D) Quantification of $VSX2^+$ BC number in the *Lhx4* null mice and control at P6, P7, P8, and P10.

(E) Compared to the control, $CASP3^+$ (green) $VSX2^+$ (red) apoptotic cells were greatly increased at P7 and the INL became thinner at P8 in *Lhx4* null mice.

(F) Quantification of the number of apoptotic cells ($n \geq 3$) in the control and *Lhx4* null retinas. Data are represented as mean ± SD. **$p < 0.01$; ***$p < 0.001$. Scale bars, 50 μm.

significantly decreased in *Lhx4* null retinas at P7 (Figures 5C, S3B, and S4A; Table S2). The above downregulated genes in *Lhx4* null retinas are involved in the biological processes related to the function of synapses. Thus, *Lhx4* likely plays an essential role in the synaptic development of BCs.

To further examine the altered transcriptome in the early stage of BC development, we compared the abnormal transcriptome of *Lhx4* null developing retinas with that of bipolar cell pseudotime (Clark et al., 2019) and found that the expression of *Bhlhe23*, *Grik1*, *Grm6*, *Scgn*, *Otor*, *Gsg1*, and *Samn1* in BCs was

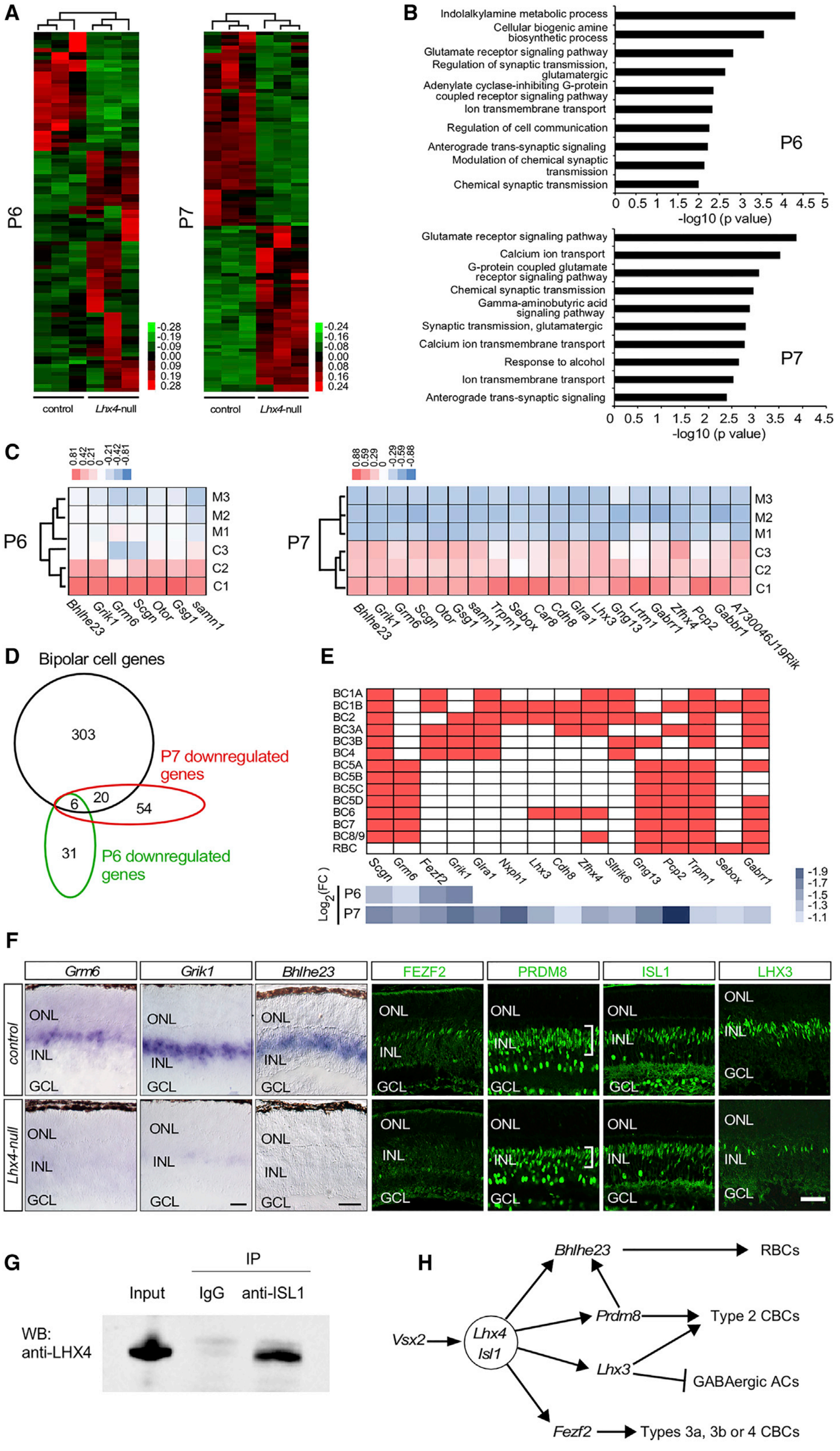

(legend on next page)

significantly decreased in *Lhx4* null retinas at P6 (Figures 5C and 5D; Table S1). Similarly, by comparing the altered transcriptome of *Lhx4* null retina with the branched expression analysis modeling (BEAM) analysis data on BCs versus photoreceptors (Clark et al., 2019), we found that *Trpm1*, *Sebox*, *Bhlhe23*, *Grm6*, *Car8*, *Cdh8*, *Glra1*, *A730046J19Rik*, *Lhx3*, *Gng13*, *Lrtm1*, *Gabrr1*, *Grik1*, *Scgn*, *Otor*, *Zfhx4*, *Pcp2*, *Gsg1*, and *Samn1* were expressed in the BCs and that their expression was significantly reduced in *Lhx4* null retinas at P7 (Figures 5C and 5D; Table S2).

To reveal bipolar-cell-type-specific phenotype analysis of the abnormal transcriptome in *Lhx4* null retinas, we compared the altered transcriptome of *Lhx4* null retinas with the bipolar cell scRNA-seq data (Shekhar et al., 2016). The expression of *Scgn* (a broad CBC marker), *Grm6* (a pan-ON-BC marker), *Fezf2* (type 1a, 1b, 3a, 3b, and 4 BCs), and *Grik1* (Type 2, 3a, 3b, and 4 BCs) was significantly reduced in the *Lhx4* null retina at P6 and P7 (Figure 5E). The expression of many other bipolar cell markers identified in the bipolar cell scRNA-seq study was also significantly decreased in the *Lhx4* null retina at P7 (Figure 5E), including *Glra1* (a pan-OFF-BC marker), *Nxph1* (type 1b and 2 BCs), *Lhx3* (type 1b, 2, and 6 BCs), *Cdh8* (type 1b, 2, 3and , 6 BCs), *Zfhx4* (type 1a, 1b, 2, 3a, 6, and 8/9 BCs), *Sltrik6* (type 1a, 1b, 2, 3b, and 4 BCs), *Gng13* (type 2 and 3b BCs and pan-ON-BCs), *Pcp 2* (type 1b and 3a BCs and pan-ON-BCs), *Trpm1* (type 1a,1b, 2, 3a, and 3b BCs and pan-ON-BCs), *Sebox* (type 1b BC and RBC), and *Gabrr1* (type 1a, 1b, 2, 3a, 3b, 5a, 5d, 6, 7, and 8/9 BCs and RBC) (Shekhar et al., 2016). Moreover, AC-type-specific phenotype on the abnormal transcriptome of the developing *Lhx4* null retina was analyzed by using the AC scRNA-seq data (Macosko et al., 2015). *Slitrk6* and *Glra1*, two significantly downregulated genes in the *Lhx4* null retina at P7, also label ACs. *Slitrk6* marks cluster 6 ACs, whereas *Glra1* marks cluster 11 ACs (Macosko et al., 2015). Immunostaining and *in situ* hybridization further confirmed that the expression of FEZF2, LHX3, *Grm6*, and *Grik1* was significantly decreased, whereas the expression of *Cacna1i* was increased in the *Lhx4* null developing retina (Figures 5E, 5F, and S4A–S4C; Tables S1 and S2). In summary, the loss of *Lhx4* results in the downregulation of genes required by RBCs and CBC subtypes.

Ablation of *Bhlhe23* Expression in the *Lhx4* Null Retina

RNA-seq results showed that one of the most downregulated genes in the *Lhx4* null retina was the bHLH TF gene *Bhlhe23* (fold change = 14.0, p = 5.00E-05 at P6 and fold change = 15.5, p = 5.00E-05 at P7; Tables S1 and S2). Previous studies have shown that *Bhlhe23* is essential for the development of RBCs (Bramblett et al., 2004). We then examined the effect of *Lhx4* inactivation on the expression of *Bhlhe23* by *in situ* hybridization at P6 when BCs are actively generated. Our results showed that the expression of *Bhlhe23* was ablated in the INL of *Lhx4* null retinas at P6 (Figure 5F). In addition, the TFs ISL1 and PRDM8 are required for the development of RBCs and CBC subtypes. The number of PRDM8-expressing BCs was reduced in *Lhx4* null retinas at P6 (Figures 5F and S4B). However, the expression of ISL1 was not affected in the BCs in *Lhx4* null retinas at P6 (Figures 5F and S4B). Previously, we have shown that inactivation of *Isl1* does not alter the expression of *Lhx4* in the retina (Elshatory et al., 2007). To investigate whether LHX4 and ISL1 could interact during BC development, we used co-immunoprecipitation and detected the LHX4-ISL1 complex in the retina at P8 (Figure 5G). Thus, LHX4 and ISL1 interact and function upstream of *Bhlhe23* and *Prdm8* during the development of BCs in the LHX4 transcriptional regulatory network of BC development (Figure 5H).

Bipolar Cell to Amacrine Cell Conversion in *Lhx4* Null Retinas

Our previous study has shown that tdTomato mimics the expression of endogenous LHX4 in the $Lhx4^{tdT/+}$ retina (Dong et al., 2019). Thus, we compared the expression of tdTomato in the *Lhx4* null ($Lhx4^{loxP/tdT}$; *Six3-Cre*) retina with that in the control ($Lhx4^{tdT/+}$ and $Lhx4^{loxP/tdT}$) retina. At P6, the number of tdTomato^{+} cells in the *Lhx4* null retina was comparable to the control. However, the number of tdTomato^{+} cells in the *Lhx4* null retina was significantly reduced compared to the control at P7, P8, and P10 (Figures 6A, 6D, S5A, and S5B). In contrast to the absence of tdTomato in PAX6+ ACs in the control, we observed that tdTomato^{+} cells expressed PAX6 and were located in the INL of *Lhx4* null retinas at P6–P10 (Figure 6A). Although the percentage of tdTomato^{+} ACs is low at P6 (3.2%) and P7 (5.9%) in

Figure 5. Loss of *Lhx4* Results in an Abnormal Transcriptome of the Developing Retina

(A) Transcriptome analysis revealed 91 differentially expressed genes at P6 and 100 differentially expressed genes at P7 in the *Lhx4* null retina compared with the control.

(B) GO enrichment for downregulated genes was performed in the *Lhx4* null retina at P6 (top image) and at P7 (bottom image).

(C) Heatmap of expression levels of downregulated BC genes in the *Lhx4* null retina at P6 and P7, determined by RNA-seq analysis.

(D) Venn diagram of downregulated genes in the *Lhx4* null retina at P6 and P7 and BC-enriched genes (Clark et al., 2019).

(E) The expression of BC-subtype-specific markers was significantly decreased in the *Lhx4* null retina. In the top image, red boxes indicate gene expression in specific BC subtypes (Shekhar et al., 2016). In the bottom image, blue-shaded boxes show the $\log_2$(fold change) in the *Lhx4* null retina compared with the control.

(F) *In situ* hybridization confirmed that the expression of *Bhlhe23* was abolished in the *Lhx4* null retina at P6, and the expression of glutamate receptor genes *Grm6* and *Grik1* was significantly decreased in the *Lhx4* null retina at P6. Immunolabeling revealed that the expression of PRDM8 in the outer edge of INL was significantly reduced in the *Lhx4* null retina at P6, whereas ISL1 expression in the INL was not affected by the loss of *Lhx4* at P6. The expression of FEZF2 and LHX3 was dramatically reduced and only very few FEZF2-expressing BCs were detected in the *Lhx4*-null retina at P6.

(G) Co-immunoprecipitation showed that LHX4 interacts with ISL1 to form a protein complex in the P8 retina.

(H) A model of the LHX4 transcriptional regulatory network in BC development: (1) loss of *Lhx4* or *Isl1* does not influence the specification of BCs, and *Lhx4* and *Isl1* likely act downstream of *Vsx2*; (2) LHX4 physically interacts with ISL1 to form a LIM protein complex; (3) LHX4-ISL1 regulates the differentiation of RBCs by *Bhlhe23*; (4) LHX4-ISL1 regulates the differentiation of type 2 CBCs and antagonizes the development of GABAergic ACs by *Lhx3*; (5) *Prdm8* is likely under the control of LHX4 during the differentiation of RBCs and type 2 CBCs; and (6) LHX4-ISL1 likely regulates the differentiation of type 3a, 3b, or 4 OFF-CBCs through *Fezf2*. Scale bars, 50 μm.

See also Figures S3 and S4.

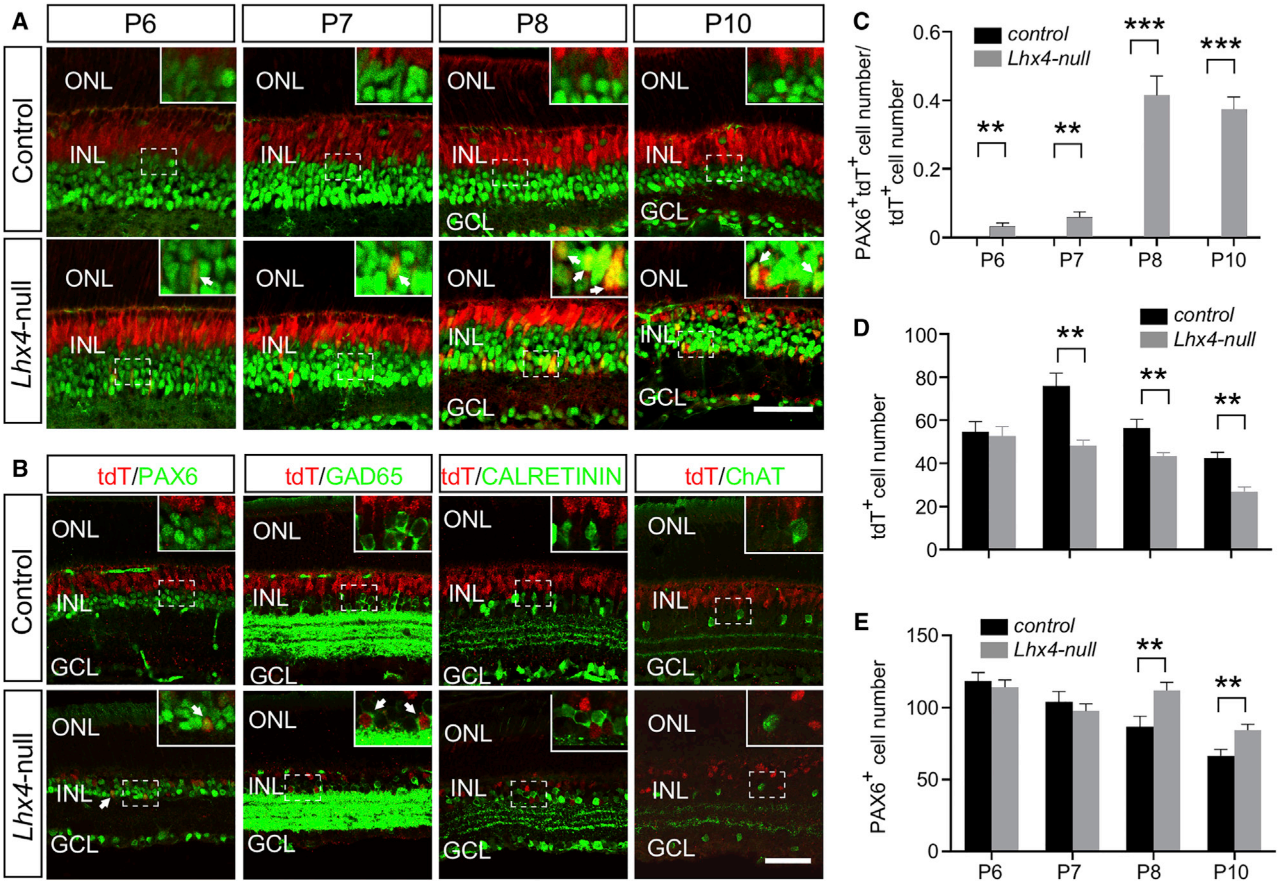

Figure 6. tdTomato-Expressing Amacrine Cells Are Detected in the *Lhx4* Null Retina

Confocal images of retinal sections were co-immunolabeled with tdTomato (red) and AC markers (green) to detect ACs originated from the Lhx4-expressing cell lineage.

(A) tdTomato$^+$ cells of the control ($Lhx4^{tdT/+}$ and $Lhx4^{tdT/loxP}$) retina were located at outer side of INL and did not express PAX6. In contrast, tdTomato$^+$/PAX6$^+$ cells were detected at the inner side of INL in the *Lhx4* null retina at P6, P7, P8, and P10.

(B) PAX6$^+$/tdTomato$^+$ and GAD65$^+$/tdTomato$^+$ cells were found in the adult *Lhx4* null retina, whereas none of the CALRETININ$^+$/tdTomato$^+$ and ChAT $^+$/tdTomato$^+$ cells was detected.

(C) The percentage of tdTomato$^+$ cells expressing PAX6 in the *Lhx4* null retina and control at P6, P7, P8, and P10.

(D) Quantification of tdTomato$^+$ cell number in the *Lhx4* null retina and control at P6, P7, P8, and P10.

(E) Quantification of PAX6$^+$ amacrine cell number in the *Lhx4* null retina and control at P6, P7, P8, and P10. Data are represented as mean ± SD. $^{**}p < 0.01$; $^{***}p < 0.001$. Scale bars, 50 μm.

See also Figure S5.

the *Lhx4* null retina, it was dramatically increased at P8 (41.5%) and P10 (37.4%) (Figure 6C). Although the number of PAX6$^+$ ACs was decreased in the adult *Lhx4* null retina (Figure 3B), the number of PAX6$^+$ ACs was not significantly affected in the *Lhx4* null retina at P6 and P7 compared to the control and was significantly increased by 29.2% at P8 and 26.4% at P10 (Figures 6A and 6E). By contrast, we did not observe the expression of tdTomato in p27kip1$^+$ Müller glia cells or CALBINDIN$^+$ horizontal cells in both control and *Lhx4* null retinas, nor did we detect any significant change in the number of Müller glia cells or horizontal cells (Figures S5A–S5D).

In the adult retina, compared to the expression of tdTomato in many BCs of the outer INL in the control, the number of cells expressing tdTomato is reduced in the INL of the *Lhx4* null retina and some of these tdTomato$^+$ cells resided at the inner side of INL and expressed PAX6 (Figure 6B). We further characterized the identity of these tdTomato$^+$ ACs in the adult retina using antibodies against GAD65, CALRETININ, and ChAT. Compared to the absence of these AC markers in the tdTomato$^+$ cells of the control, tdTomato expression was seen in a subset of GAD65$^+$ ACs of the *Lhx4* null retina (Figure 6B, arrows) but not in CALRETININ$^+$ and ChAT$^+$ ACs of the *Lhx4* null retina (Figure 6B). In summary, the loss of *Lhx4* results in cell fate conversion of some BCs to ACs, especially those of the GABAergic subtype.

Overexpression of *Lhx4* Promotes Bipolar Cell Genesis

To determine if the expression of *Lhx4* promoted bipolar cell differentiation, we overexpressed LHX4 in wild-type retinas at P0 by electroporation and collected the retinas at P10. About 89.4% of GFP$^+$ INL cells in the pCAGIG-*Lhx4* electroporated

retina were identified with VSX2^{+} BCs, whereas only about 64.5% of GFP^{+} INL cells in the pCAGIG electroporated control retina were identified with VSX2^{+} BCs (Figures 7A and 7D). It suggests that overexpression of *Lhx4* could drive the generation of VSX2^{+} BCs. However, the ratio of PRDM8^{+}/GFP^{+} cells to GFP^{+} INL cells and the ratio of FEZF2^{+}/GFP^{+} cells to GFP^{+} INL cells were not significantly altered in the pCAGIG-*Lhx4*-electroporated retina compared to the pCAGIG electroporated retina (Figures 7B–7D). Previous studies have shown that LIM proteins recruit other LIM proteins and TFs to regulate neuronal development (Kim et al., 2017; Thaler et al., 2002). LHX3 interacts with ISL1 to drive the generation of the motor neuron in the spinal cord and LHX3 forms a LIM protein complex with ISL1 and Tgfb1i1 to antagonize the development of GABAergic ACs (Kim et al., 2017; Thaler et al., 2002). LHX4 likely requires the cooperation of other TFs, such as ISL1, to drive the expression of PRDM8 and FEZF2 and further differentiation of BCs.

Overexpression of *Bhlhe23* Rescues RBCs in the *Lhx4* Null Retina

Bhlhe23 is necessary for the differentiation of RBCs, and the loss of *Bhlhe23* results in the near absence of RBCs in the retina (Bramblett et al., 2004). Because the loss of *Lhx4* results in absence of RBCs and ablates the expression of *Bhlhe23* in the *Lhx4* null retina (Figures 2F and 5F), we examined whether the overexpression of *Bhlhe23* could function downstream of *Lhx4* to rescue RBCs in the *Lhx4* null retina. We electroporated pCAGIG-*Bhlhe23* and pCAGIG vectors into *Lhx4* null retinas at P0 and collected retinas for analysis at P10. Co-immunolabeling revealed GFP^{+}/PRKCA^{+} RBCs in the pCAGIG-*Bhlhe23*-electroporated *Lhx4* null retina but not in the pCAGIG-electroporated *Lhx4* null retina (Figures 7E and 7F). Therefore, *Bhlhe23* could rescue RBC differentiation deficiency in the absence of *Lhx4*.

DISCUSSION

The LIM-HD TFs regulate gene expression by direct binding to the regulatory sequences by its DNA-binding HD or by interacting with other TFs or both. In the spinal cord, the overlapping expression and combinatorial role of ISL1, LHX3, and LHX4 in subtype assignment of motor neurons have been revealed (Pfaff et al., 1996; Sharma et al., 1998). ISL1 and LHX3 are shown to form a transcriptional activating complex, and the ISL1/LHX3 complex upregulates the expression of ISL2 and LHX4, which function redundantly with ISL1 and LHX3 (Lee et al., 2012). In the retina, ISL1, LHX3, and LHX4 are all expressed in BCs, and the expression of ISL1 in RBCs and ON-CBCs partially overlaps that of LHX3 and LHX4 (Balasubramanian et al., 2014; Elshatory et al., 2007). Targeted deletion of *Isl1* results in the loss of RBCs and in the great reduction of ON- and OFF-CBCs, including type 2 BCs. Interestingly, the expression of LHX4 is not affected in the *Isl1* null retina, whereas the expression of *Lhx3* is nearly ablated (Elshatory et al., 2007). Likewise, the loss of *Lhx4* does not affect ISL1 expression in developing BCs, whereas LHX3 expression is greatly reduced (Figures 5C, 5F, and S4A–S4C), suggesting that *Isl1* and *Lhx4* function in parallel and upstream of *Lhx3* in regulating BC development. Co-immunoprecipitation shows LHX4 physically interacts with ISL1 to form a LIM protein complex in the retina (Figure 5G), suggesting that the LHX4-ISL1 complex could regulate the expression of *Lhx3* in the retina. A previous study has shown that *Lhx3* promotes the development of BHLHB5^{+} type 2 BCs and antagonizes the development of GABAergic ACs (Kim et al., 2017). Consistently, our finding reveals that the loss of *Lhx4* results in a cell fate switch from some BCs to GABAergic ACs (Figures 6A and 6B). Therefore, LHX4 and ISL1 positively regulate the expression of LHX3 to drive the fate choice of type 2 BCs over the GABAergic ACs.

The specification of BCs and the differentiation of BC subtypes depend on the coordinated expression of many TFs. VSX2 is required for the specification of BCs, and the loss of *Vsx2* results in the absence of BCs in mice (Burmeister et al., 1996). The *Lhx4* null mutation does not affect VSX2 expression at P6 (Figure 4C), indicating that VSX2 expression does not depend on LHX4. In addition, we have shown that the loss of *Lhx4* does not affect the number of BCs at P6. Rather, in the absence of *Lhx4*, a significant loss of nascent BCs occurs starting at P7 (Figure 4C). Similarly, the specification of BCs is not affected at P6 and the number of BCs is significantly reduced at P7 in the *Isl1* null retina (Elshatory et al., 2007), implying that LHX4 and ISL1 are dispensable for the initial generation of BCs but are required for the differentiation and survival of BC subtypes.

Published studies have revealed that many TF genes acts coordinately in the differentiation of RBCs and CBC subtypes. *Bhlhe23* is expressed in all developing RBCs and is required for RBC differentiation. RBCs are mostly gone in *Bhlhe23* null mice, accounting for the loss of the scotopic ERG b-wave (Bramblett et al., 2004). The phenotype of RBC loss in *Bhlhe23* null mice strongly resembles that in mice null for *Isl1* or *Lhx4*. Strikingly, the expression of *Bhlhe23* mRNA is absent in the *Lhx4* null retina (Figure 5F) and is markedly reduced in the *Isl1* null retina (Elshatory et al., 2007), indicating that LHX4-ISL1 regulates the differentiation of RBCs by *Bhlhe23* (Figure 5H). PRDM8 functions upstream of *Bhlhe23* during BC development and is essential for the differentiation and survival of RBCs and type 2 OFF-CBCs (Jung et al., 2015). The nearly complete loss of RBCs in *Prdm8* null mice resembles those phenotypes observed in mice null for *Bhlhe23*, *Isl1*, or *Lhx4*. In this study, we have demonstrated that PRDM8 expression is significantly reduced in the *Lhx4* null retina (Figures 5F and S4B), suggesting that *Lhx4* might regulate *Prdm8* expression in the RBCs. Taken together, these results suggest a *Vsx2*→*Lhx4-Isl1*→*Prdm8*→*Bhlhe23* regulatory pathway of RBC development (Figure 5H).

Presently, how each of the CBC subtype identities is specified from bipolar precursors is not well understood. PRDM8 is essential for the differentiation and survival of RBCs and type 2 OFF-CBCs in the developing and adult mouse retinas. *Fezf2* is expressed in the type 1a, 1b, 3a, 3b, and 4 OFF-CBCs at P17 (Shekhar et al., 2016) and is required for the differentiation of OFF-CBCs and functional maturation of ON-CBCs (Suzuki-Kerr et al., 2018). In the *Fezf2* null retina, the total number of OFF-CBCs is significantly reduced, whereas the number of TACR3^{+} type 1a, 1b, and 2 OFF-CBCs is not affected (Shekhar et al., 2016; Suzuki-Kerr et al., 2018). In this study, we show that targeted deletion of *Lhx4* results in the nearly complete

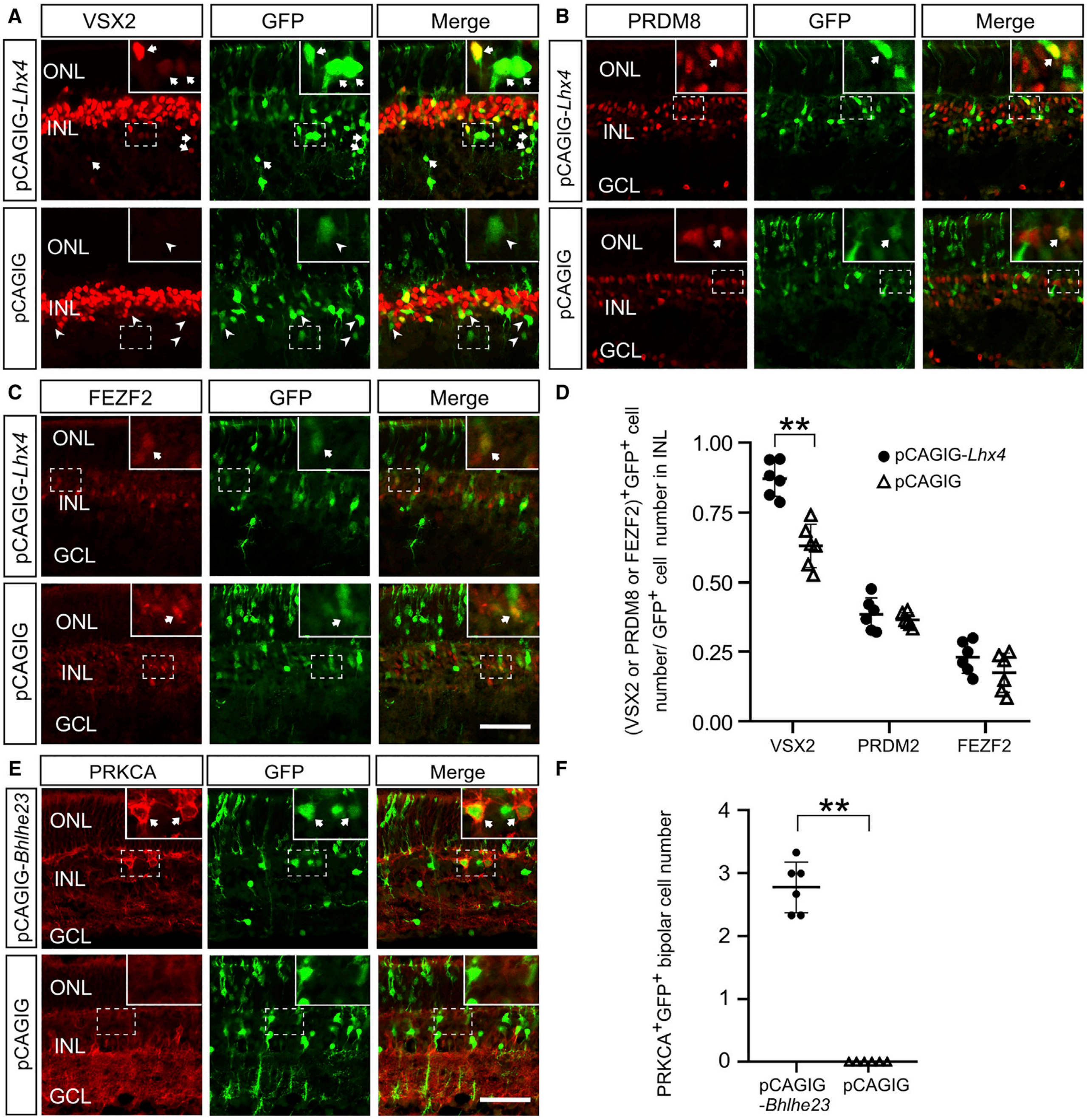

Figure 7. Overexpression of *Lhx4* Drives BC Genesis, and Overexpression of *Bhlhle23* Rescues RBCs in the Absence of *Lhx4*

Six control mice and six *Lhx4* null mice were used in each experiment, and each point in the graph represents the average cell count of three images per mouse.

(A) Confocal images showed the co-expression of VSX2 and GFP in the pCAGIG-*Lhx4*- and pCAGIG-electroporated retina. Arrow, GFP$^+$/VSX2$^+$ cells; arrowhead, GFP$^+$/VSX2$^-$ cells.

(B) The expression of PRDM8 and GFP in the pCAGIG-*Lhx4*- and pCAGIG-electroporated retinas.

(C) The expression of FEZF2 and GFP in the pCAGIG-*Lhx4*- and pCAGIG-electroporated retina.

(D) The percentage of GFP$^+$ cells in the INL expressing VSX2, PRDM8, and FEZF2 in the pCAGIG-*Lhx4*- and pCAGIG-electroporated retinas.

(E) GFP$^+$/PRKCA$^+$ cells were detected in the pCAGIG-*Bhlhe23*-electroporated *Lhx4* null retina but not in the pCAGIG-electroporated *Lhx4* null retina.

(F) Quantification of GFP$^+$/PRKCA$^+$ cells in the pCAGIG-*Bhlhe23*-electroporated *Lhx4* null retina and the pCAGIG-electroporated *Lhx4* null retina. Data are represented as mean ± SD. **p < 0.01. Scale bars, 50 μm.

CellPress
OPEN ACCESS

loss of type 3a, 3b, and 4 OFF-CBCs and in the reduction of type 2 OFF-CBCs, indicating LHX4's role in specifying these CBC subtypes. Similarly, type 2, 3a, and 3b OFF-CBCs are significantly decreased in *Isl1*-null retina (Elshatory et al., 2007). The expression of PRDM8 is significantly downregulated in the *Lhx4* null retina (Figures 5F and S4B), suggesting that LHX4-ISL1 could regulate the differentiation of type 2 OFF-CBCs through *Prdm8* (Figure 5H). Similarly, our data show that the expression of *Fezf2* is severely decreased in the *Lhx4* null retina (Figures 5E, 5F, and S4A–S4C), suggesting that LHX4-ISL1 might regulate the differentiation of type 3a, 3b, or 4 OFF-CBCs and the function of ON-CBCs by *Fezf2* (Figure 5H).

Lhx4 is involved in the regulation of differentiation and development of the pituitary gland, and *Lhx4* null mice exhibit incomplete pituitary gland development. Heterozygous mutations in *LHX4* are known to cause pituitary hormone deficiency diseases, such as syndromic short stature, and a homozygous mutation in *LHX4* results in death within 1 week after birth (Cohen et al., 2017; Gregory et al., 2015; Machinis et al., 2001). However, no mutation in *LHX4* has been identified to be associated with CSNB in humans. Our ERG studies of *Lhx4* null mice have revealed that the scotopic b-wave is absent in the *Lhx4* null mice. This phenotype resembles the symptoms observed in patients with CSNB and strongly argues for *LHX4* as a candidate gene of CSNB in humans.

STAR★METHODS

Detailed methods are provided in the online version of this paper and include the following:

- KEY RESOURCES TABLE
- RESOURCE AVAILABILITY
  - Lead Contact
  - Materials Availability
  - Data and Code Availability
- EXPERIMENTAL MODEL AND SUBJECT DETAILS
- METHOD DETAILS
  - H&E staining, immunolabeling and *in situ* hybridization
  - Electroretinogram
  - RNA-Sequencing (RNA-Seq)
  - Co-immunoprecipitation and western blot
  - Subretinal DNA electroporation
  - Droplet Digital PCR (ddPCR) quantification
- QUANTIFICATION AND STATISTICAL ANALYSIS

SUPPLEMENTAL INFORMATION

Supplemental Information can be found online at https://doi.org/10.1016/j.celrep.2020.108144.

ACKNOWLEDGMENTS

We thank Drs. Amy Kiernan, Richard Libby, Patricia White, and the members of Gan laboratory for their insightful discussions. This research was supported by a National Institutes of Health grant (EY026614) to L. Gan, the Research to Prevent Blindness challenge grant to the Department of Ophthalmology, and the P30 Center Core grant (EY01319F) to the Center for Visual Science at the University of Rochester.

AUTHOR CONTRIBUTIONS

L. Gan and G.L. conceived and designed experiments; X.D., H.Y., X.Z., X.X., L. Guo, M.X., and W.Z. performed the experiments. X.D. and D.Y. analyzed the data; X.D., G.L., and L. Gan wrote the paper.

DECLARATION OF INTERESTS

The authors declare no competing interests.

Received: August 19, 2019
Revised: July 13, 2020
Accepted: August 21, 2020
Published: September 15, 2020

REFERENCES

Balasubramanian, R., Bui, A., Ding, Q., and Gan, L. (2014). Expression of LIM-homeodomain transcription factors in the developing and mature mouse retina. Gene Expr. Patterns *14*, 1–8.

Blackshaw, S., Harpavat, S., Trimarchi, J., Cai, L., Huang, H., Kuo, W.P., Weber, G., Lee, K., Fraioli, R.E., Cho, S.H., et al. (2004). Genomic analysis of mouse retinal development. PLoS Biol. *2*, E247.

Bramblett, D.E., Pennesi, M.E., Wu, S.M., and Tsai, M.J. (2004). The transcription factor Bhlhb4 is required for rod bipolar cell maturation. Neuron *43*, 779–793.

Buenaventura, D.F., Corseri, A., and Emerson, M.M. (2019). Identification of Genes With Enriched Expression in Early Developing Mouse Cone Photoreceptors. Invest. Ophthalmol. Vis. Sci. *60*, 2787–2799.

Burmeister, M., Novak, J., Liang, M.Y., Basu, S., Ploder, L., Hawes, N.L., Vidgen, D., Hoover, F., Goldman, D., Kalnins, V.I., et al. (1996). Ocular retardation mouse caused by Chx10 homeobox null allele: impaired retinal progenitor proliferation and bipolar cell differentiation. Nat. Genet. *12*, 376–384.

Clark, B.S., Stein-O'Brien, G.L., Shiau, F., Cannon, G.H., Davis-Marcisak, E., Sherman, T., Santiago, C.P., Hoang, T.V., Rajaii, F., James-Esposito, R.E., et al. (2019). Single-Cell RNA-Seq Analysis of Retinal Development Identifies NFI Factors as Regulating Mitotic Exit and Late-Born Cell Specification. Neuron *102*, 1111–1126.e1115.

Cohen, E., Maghnie, M., Collot, N., Leger, J., Dastot, F., Polak, M., Rose, S., Touraine, P., Duquesnoy, P., Tauber, M., et al. (2017). Contribution of LHX4 Mutations to Pituitary Deficits in a Cohort of 417 Unrelated Patients. J. Clin. Endocrinol. Metab. *102*, 290–301.

de Melo, J., and Blackshaw, S. (2018). In Vivo Electroporation of Developing Mouse Retina. Methods Mol. Biol. *1715*, 101–111.

Deng, M., Pan, L., Xie, X., and Gan, L. (2010). Requirement for Lmo4 in the vestibular morphogenesis of mouse inner ear. Dev. Biol. *338*, 38–49.

Dong, X., Xie, X., Guo, L., Xu, J., Xu, M., Liang, G., and Gan, L. (2019). Generation and characterization of Lhx4(tdT) reporter knock-in and Lhx4(loxP) conditional knockout mice. Genesis *57*, e23328.

Elshatory, Y., Everhart, D., Deng, M., Xie, X., Barlow, R.B., and Gan, L. (2007). Islet-1 controls the differentiation of retinal bipolar and cholinergic amacrine cells. J. Neurosci. *27*, 12707–12720.

Euler, T., Haverkamp, S., Schubert, T., and Baden, T. (2014). Retinal bipolar cells: elementary building blocks of vision. Nat. Rev. Neurosci. *15*, 507–519.

Feng, L., Xie, X., Joshi, P.S., Yang, Z., Shibasaki, K., Chow, R.L., and Gan, L. (2006). Requirement for Bhlhb5 in the specification of amacrine and cone bipolar subtypes in mouse retina. Development *133*, 4815–4825.

Ghosh, K.K., Bujan, S., Haverkamp, S., Feigenspan, A., and Wässle, H. (2004). Types of bipolar cells in the mouse retina. J. Comp. Neurol. *469*, 70–82.

Gregory, L.C., Humayun, K.N., Turton, J.P., McCabe, M.J., Rhodes, S.J., and Dattani, M.T. (2015). Novel Lethal Form of Congenital Hypopituitarism Associated With the First Recessive LHX4 Mutation. J. Clin. Endocrinol. Metab. *100*, 2158–2164.

Hack, I., Peichl, L., and Brandstätter, J.H. (1999). An alternative pathway for rod signals in the rodent retina: rod photoreceptors, cone bipolar cells, and the localization of glutamate receptors. Proc. Natl. Acad. Sci. USA *96*, 14130–14135.

Hatakeyama, J., Tomita, K., Inoue, T., and Kageyama, R. (2001). Roles of homeobox and bHLH genes in specification of a retinal cell type. Development *128*, 1313–1322.

Haverkamp, S., and Wässle, H. (2000). Immunocytochemical analysis of the mouse retina. J. Comp. Neurol. *424*, 1–23.

Haverkamp, S., Ghosh, K.K., Hirano, A.A., and Wässle, H. (2003). Immunocytochemical description of five bipolar cell types of the mouse retina. J. Comp. Neurol. *455*, 463–476.

Haverkamp, S., Specht, D., Majumdar, S., Zaidi, N.F., Brandstätter, J.H., Wasco, W., Wässle, H., and Tom Dieck, S. (2008). Type 4 OFF cone bipolar cells of the mouse retina express calsenilin and contact cones as well as rods. J. Comp. Neurol. *507*, 1087–1101.

Huang, L., Hu, F., Feng, L., Luo, X.J., Liang, G., Zeng, X.Y., Yi, J.L., and Gan, L. (2014). Bhlhb5 is required for the subtype development of retinal amacrine and bipolar cells in mice. Dev. Dyn. *243*, 279–289.

Jung, C.C., Atan, D., Ng, D., Ploder, L., Ross, S.E., Klein, M., Birch, D.G., Diez, E., and McInnes, R.R. (2015). Transcription factor PRDM8 is required for rod bipolar and type 2 OFF-cone bipolar cell survival and amacrine subtype identity. Proc. Natl. Acad. Sci. USA *112*, E3010–E3019.

Kim, Y., Lim, S., Ha, T., Song, Y.H., Sohn, Y.I., Park, D.J., Paik, S.S., Kim-Kaneyama, J.R., Song, M.R., Leung, A., et al. (2017). The LIM protein complex establishes a retinal circuitry of visual adaptation by regulating Pax6 α-enhancer activity. eLife *6*, e21303.

Kuleshov, M.V., Jones, M.R., Rouillard, A.D., Fernandez, N.F., Duan, Q., Wang, Z., Koplev, S., Jenkins, S.L., Jagodnik, K.M., Lachmann, A., et al. (2016). Enrichr: a comprehensive gene set enrichment analysis web server 2016 update. Nucleic Acids Res. *44*, W90–W97.

Lee, S., Cuvillier, J.M., Lee, B., Shen, R., Lee, J.W., and Lee, S.K. (2012). Fusion protein Isl1-Lhx3 specifies motor neuron fate by inducing motor neuron genes and concomitantly suppressing the interneuron programs. Proc. Natl. Acad. Sci. USA *109*, 3383–3388.

Liu, Y., Fan, M., Yu, S., Zhou, Y., Wang, J., Yuan, J., and Qiang, B. (2002). cDNA cloning, chromosomal localization and expression pattern analysis of human LIM-homeobox gene LHX4. Brain Res. *928*, 147–155.

Livne-Bar, I., Pacal, M., Cheung, M.C., Hankin, M., Trogadis, J., Chen, D., Dorval, K.M., and Bremner, R. (2006). Chx10 is required to block photoreceptor differentiation but is dispensable for progenitor proliferation in the postnatal retina. Proc. Natl. Acad. Sci. USA *103*, 4988–4993.

Machinis, K., Pantel, J., Netchine, I., Léger, J., Camand, O.J.A., Sobrier, M.-L., Dastot-Le Moal, F., Duquesnoy, P., Abitbol, M., Czernichow, P., and Amselem, S. (2001). Syndromic short stature in patients with a germline mutation in the LIM homeobox LHX4. Am. J. Hum. Genet. *69*, 961–968.

Macosko, E.Z., Basu, A., Satija, R., Nemesh, J., Shekhar, K., Goldman, M., Tirosh, I., Bialas, A.R., Kamitaki, N., Martersteck, E.M., et al. (2015). Highly Parallel Genome-wide Expression Profiling of Individual Cells Using Nanoliter Droplets. Cell *161*, 1202–1214.

Mataruga, A., Kremmer, E., and Müller, F. (2007). Type 3a and type 3b OFF cone bipolar cells provide for the alternative rod pathway in the mouse retina. J. Comp. Neurol. *502*, 1123–1137.

Matsuda, T., and Cepko, C.L. (2004). Electroporation and RNA interference in the rodent retina in vivo and in vitro. Proc. Natl. Acad. Sci. USA *101*, 16–22.

Pan, L., Yang, Z., Feng, L., and Gan, L. (2005). Functional equivalence of Brn3 POU-domain transcription factors in mouse retinal neurogenesis. Development *132*, 703–712.

Pfaff, S.L., Mendelsohn, M., Stewart, C.L., Edlund, T., and Jessell, T.M. (1996). Requirement for LIM homeobox gene Isl1 in motor neuron generation reveals a motor neuron-dependent step in interneuron differentiation. Cell *84*, 309–320.

Rice, D.S., and Curran, T. (2000). Disabled-1 is expressed in type AII amacrine cells in the mouse retina. J. Comp. Neurol. *424*, 327–338.

Sharma, K., Sheng, H.Z., Lettieri, K., Li, H., Karavanov, A., Potter, S., Westphal, H., and Pfaff, S.L. (1998). LIM homeodomain factors Lhx3 and Lhx4 assign subtype identities for motor neurons. Cell *95*, 817–828.

Shekhar, K., Lapan, S.W., Whitney, I.E., Tran, N.M., Macosko, E.Z., Kowalczyk, M., Adiconis, X., Levin, J.Z., Nemesh, J., Goldman, M., et al. (2016). Comprehensive Classification of Retinal Bipolar Neurons by Single-Cell Transcriptomics. Cell *166*, 1308–1323.e1330.

Sheng, H.Z., Moriyama, K., Yamashita, T., Li, H., Potter, S.S., Mahon, K.A., and Westphal, H. (1997). Multistep control of pituitary organogenesis. Science *278*, 1809–1812.

Suzuki-Kerr, H., Iwagawa, T., Sagara, H., Mizota, A., Suzuki, Y., and Watanabe, S. (2018). Pivotal roles of Fezf2 in differentiation of cone OFF bipolar cells and functional maturation of cone ON bipolar cells in retina. Exp. Eye Res. *171*, 142–154.

Thaler, J.P., Lee, S.K., Jurata, L.W., Gill, G.N., and Pfaff, S.L. (2002). LIM factor Lhx3 contributes to the specification of motor neuron and interneuron identity through cell-type-specific protein-protein interactions. Cell *110*, 237–249.

Tomita, K., Moriyoshi, K., Nakanishi, S., Guillemot, F., and Kageyama, R. (2000). Mammalian achaete-scute and atonal homologs regulate neuronal versus glial fate determination in the central nervous system. EMBO J. *19*, 5460–5472.

Trapnell, C., Roberts, A., Goff, L., Pertea, G., Kim, D., Kelley, D.R., Pimentel, H., Salzberg, S.L., Rinn, J.L., and Pachter, L. (2012). Differential gene and transcript expression analysis of RNA-seq experiments with TopHat and Cufflinks. Nat. Protoc. *7*, 562–578.

Völgyi, B., Deans, M.R., Paul, D.L., and Bloomfield, S.A. (2004). Convergence and segregation of the multiple rod pathways in mammalian retina. J. Neurosci. *24*, 11182–11192.

Wang, M.M., Janz, R., Belizaire, R., Frishman, L.J., and Sherry, D.M. (2003). Differential distribution and developmental expression of synaptic vesicle protein 2 isoforms in the mouse retina. J. Comp. Neurol. *460*, 106–122.

Zeitz, C., Robson, A.G., and Audo, I. (2015). Congenital stationary night blindness: an analysis and update of genotype-phenotype correlations and pathogenic mechanisms. Prog. Retin. Eye Res. *45*, 58–110.

STAR★METHODS

KEY RESOURCES TABLE

REAGENT or RESOURCE	SOURCE	IDENTIFIER
Antibodies		
Rabbit anti-activated caspase-3	R and D Systems	Cat# AF835; RRID: AB_2243952
Goat anti-ChAT	Millipore	Cat# AB144P; RRID: AB_2079751
Mouse anti-PAX6	DSHB	Cat# Pax6; RRID: AB_528427
Rabbit anti-PAX6	Covance Research Products Inc.	Cat# PRB-278P; RRID: AB_291612
Mouse anti-GAD65	BD Biosciences	Cat# 559931; RRID: AB_397380
Mouse anti-ISL1/2	DSHB	Cat# 39.4D5; RRID: AB_2314683
Rabbit anti-ISL1	Abcam	Cat# ab20670; RRID: AB_881306
Rabbit anti-RCVRN	Millipore	Cat# AB5585; RRID: AB_2253622
Rabbit anti-vGlut1	Millipore	Cat# AB5905; RRID: AB_2301751
Rabbit anti-SV2B	Synaptic Systems	Cat# 119 102; RRID: AB_887803
Mouse anti-GNAO1	Millipore	Cat# MAB3073; RRID: AB_94671
Mouse anti-PRKCA	Millipore	Cat# 05-154; RRID: AB_2284233
Guinea pig anti-LHX4	gift of T.M. Jessell, Columbia University	N/A
Rabbit anti-LHX4	Proteintech	Cat# 11183-1-AP; RRID: AB_2858194
Rabbit anti-HCN4	Alomone Labs	Cat# APC-052; RRID: AB_2039906
Mouse anti-KCNIP3	Millipore	Cat# 05-756; RRID: AB_309969
Mouse anti-PRKAR2B	BD Biosciences	Cat# 610625; RRID: AB_397957
Guinea pig anti-PRDM8	Sarah Ross Lab University of Pittsburgh	Cat# GP-Prdm8 228-457; RRID: AB_266545
Bacterial and Virus Strains		
One Shot TOP10 Chemically Competent *E. coli*	Invitrogen	Cat# C404003
Chemicals, Peptides, and Recombinant Proteins		
Paraformaldehyde	Sigma	Cat# P6148
Diethyl pyrocarbonate	Sigma	Cat# D5758
Triton X-100	Sigma	Cat# T8787
Tween-20	Sigma	Cat# P-8341
Formamide	Invitrogen	Cat# 15515-026
Critical Commercial Assays		
RNeasy Mini Kit	QIAGEN	Cat# 74106
TruSeq RNA Sample Prep Kit	Illumina	Cat# RS-122-2001
iScript cDNA Synthesis Kit	Bio-Rad	Cat# 1708890
Deposited Data		
RNA-seq data of P6 control and Lhx4-null mice	This Paper	GEO: GSE126942
RNA-seq data of P7 control and Lhx4-null mice	This Paper	GEO: GSE127771
Experimental Models: Organisms/Strains		
Lhx4loxP/loxP mouse strains	Dong et al., 2019	N/A
Lhx4tdT/+ mouse strains	Dong et al., 2019	N/A
Oligonucleotides		
Primer: *Lhx4^{loxP}* Forward: TGA AGC TAT CAG GAG GCC TAG AGT	Dong et al., 2019	N/A

(*Continued on next page*)

Continued

REAGENT or RESOURCE	SOURCE	IDENTIFIER
Primer: *Lhx4*^{loxP} Reverse: AGC ATG GCC AGC TCT GCT TAC CGT	Dong et al., 2019	N/A
Primer: *Lhx4* ^{tdT} Forward: CAC GCT GAT CTA CAA GGT GAA GA	Dong et al., 2019	N/A
Primer: *Lhx4* ^{tdT} Reverse: ACC TTG AAG CGC ATG AAC TCT	Dong et al., 2019	N/A
Recombinant DNA		
pbks-Beta4-1.5	Bramblett et al., 2004	N/A
pCAGIG	Addgene	Cat# 11159
pCAGIG-*Lhx4*	This paper	N/A
pCAGIG-*Bhlhe23*	This paper	N/A
Software and Algorithms		
Tophat	Trapnell et al., 2012	RRID: SCR_013035
Cufflinks	Trapnell et al., 2012	RRID: SCR_014597
Graphpad Prism 6	GraphPad	RRID:SCR_000306
QuantaSoft Analysis Pro	Bio-Rad	N/A
Enrichr	Kuleshov et al., 2016	http://amp.pharm.mssm.edu/Enrichr/
Other		
BM-purple AP substrate	Roche	Cat#11442074001
Protein A/G agarose beads	Santa Cruz Biotechnology	Cat# sc-2003; RRID:AB_10201400
2x Laemmli Sample Buffer	Bio-Rad	Cat# 1610737
VeriBlot for IP Dectection Reagent	Abcam	Cat# ab131366
QX200 EvaGreen Digital PCR Supermix	Bio-Rad	Cat# 186-4033
Tissue-Tek O.C.T. Compound	Tissue-Tek	Cat# 4583
RIPA buffer	ThermoFisher	Cat# 89900
T7 RNA polymerase	Ambion	Cat# 2085
T3 RNA polymerase	Promega	Cat# P4024
RNase Inhibitor	Ambion	Cat# 2682
DIG RNA Labeling Mix	Roche	Cat# 1277073

RESOURCE AVAILABILITY

Lead Contact

Further information and requests for resources and reagents should be directed to and will be fulfilled by the Lead Contact, Lin Gan (ligan@augusta.edu).

Materials Availability

All reagents generated in this study are available from the Lead Contact with a completed Materials Transfer Agreement.

Data and Code Availability

The accession number for the sequencing data reported in this paper is GEO: GSE126942 and GEO: GSE127771.

EXPERIMENTAL MODEL AND SUBJECT DETAILS

The generation of *Lhx4* conditional knockout and tdTomato knock-in mice were previously described (Dong et al., 2019). The following PCR primers were used to identify the *Lhx4*^{loxP} conditional knockout allele: 5′- TGA AGC TAT CAG GAG GCC TAG AGT -3′ and 5′- AGC ATG GCC AGC TCT GCT TAC CGT -3′. The PCR primers used to identify the *Lhx4*^{tdT} knock-in allele were 5′- CAC GCT GAT CTA CAA GGT GAA GA -3′ and 5′- ACC TTG AAG CGC ATG AAC TCT -3′. University Committee of Animal Resources at the University of Rochester, August University, Hangzhou Normal University, and Wenzhou Medical University approved all animal procedures in this study. Animals of both sexes in the development (P6, P7, P8, and P10) and at adult (6-8 weeks old) were used in the experiment.

METHOD DETAILS

H&E staining, immunolabeling and *in situ* hybridization

For H&E staining, eyecups were embedded in paraffin after fixation and dehydration, and sectioned at a thickness of 8 μm. H&E staining was performed as previously described (Pan et al., 2005).

Immunofluorescence labeling was performed as previously described (Huang et al., 2014). In brief, eyes dissected from mice were fixed in 4% PFA in PBS for 1-2h at 4°C, and the eyecups were obtained by removing cornea and lens. Eyecups were cryoprotected in 30% sucrose in PBS and embedded in OCT compound (TissureTek). Cryosections were cut at a thickness of 18 μm. After blocking in 10% horse serum in PBST (0.3% Triton X-100 in PBS, pH7.3), sections were incubated with primary antibodies overnight at 4°C. After four times washing with PBST, sections were incubated with Alexa fluor-conjugated secondary antibodies for 1.5h at room temperature. They were washed three times with PBS for 5 minutes each and were counter-stained with 4′,6-diamidino-2-phenylindole (DAPI) before mounting with coverslips. Images were acquired with a Zeiss LSM 510 confocal microscope. The following antibodies were used for immunofluorescence: rabbit anti-activated caspase-3 (1:200, R and D Systems Cat# AF835, RRID: AB_2243952), goat anti-ChAT (1:50, Millipore Cat# AB144P, RRID: AB_2079751), mouse anti-PAX6 (1:200, DSHB Cat# Pax6, RRID: AB_528427), rabbit anti-PAX6 (1:200, Covance Research Products Inc. Cat# PRB-278P, RRID: AB_291612), mouse anti-GAD65 (1:200, BD Biosciences Cat# 559931, RRID: AB_397380), mouse anti-ISL1/2 (1:200, DSHB Cat# 39.4D5, RRID: AB_2314683), rabbit anti-RCVRN (1:500, Millipore Cat# AB5585, RRID: AB_2253622), rabbit anti-vGlut1 (1:1,000, Millipore Cat# AB5905, RRID: AB_2301751), rabbit anti-SV2B (1:500, Synaptic Systems Cat# 119 102, RRID:AB_887803), mouse anti-GNAO1 (1:500, Millipore Cat# MAB3073, RRID: AB_94671), mouse anti-PRKCA (1:500, Millipore Cat# 05-154, RRID: AB_2284233), guinea pig anti-LHX4 (1:250, gift of T.M. Jessell, Columbia University), rabbit anti-HCN4 (1:500, Alomone Labs Cat# APC-052, RRID: AB_2039906), mouse anti-KCNIP3 (1:1,000, Millipore Cat# 05-756, RRID: AB_309969), mouse anti-PRKAR2B (1:1,000, BD Biosciences Cat# 610625, RRID: AB_397957), and guinea pig anti-PRDM8 (1:2,000, Sarah Ross Lab University of Pittsburgh Cat# GP-Prdm8 228-457, RRID: AB_266545).

Section *in situ* hybridization was performed as previously described (Deng et al., 2010). In brief, eyecups were prepared as described in immunolabeling, cryoprotected in 20% sucrose in PBS (DEPC) and embedded in OCT compound (TissureTek). Cryosections were cut at a thickness of 18 μm. Sections were post-fixed in the 4% PFA in PBS for 5 min. Then, sections were rinsed with PBS and incubated in 10 μg/ml proteinase K for 2 min. After washing with PBS, sections were incubated in 0.1 M Triethanolamine for 10 min and re-fixed in 4% PFA for 5 min. Sections were washed with PBS, and 200 μl hybridization solution was added on each slide and pre-hybridized for 1 hour at 55°C. The hybridization probe (0.2 μg probe to 200 μl Hybridization Solution) was added on the slide overnight at 55°C. The sections were washed in washing solution (50% formamide, 2X SSC) at 65°C for 30 min. Then, sections were washed in RNase buffer at 37°C for 10 min, and incubated in 20 μg/ml RNaseA in RNase buffer at 37°C for 30 min. After washing with RNase buffer, washing solution, 2X SSC, 0.1xSSC and PBT (PBS plus 0.1%Tween-20), sections were blocked for 1 hour at RT with 10% heat-inactivated goat serum in PBT. Then, sections were incubated with alkaline phosphatase-coupled anti-digoxigenin antibody diluted at 1:5000 in PBT with 1% goat serum at 4°C overnight. After washing with NTMT four times, detection was done with BM-purple AP substrate (Roche #11442074001). Digoxigenin-labeled RNA probes were synthesized *in vitro* from cDNA for *Grm6*, *Grik1* and *Cacnca1i*. The following reaction was set up for *in vitro* transcription: 1μl linearized DNA template (1 μg/μl), 4 μl 5x transcription buffer, 2 μl DTT (100mM), 1 μl RNase inhibitor, 2 μl DIG RNA labeling mix, 1 μl T7 or T3 RNA polymerase, 9 μl DEPC water, incubate at 37°C for 2 hours. The *Bhlhe23* riboprobe construct, Beta4-1.5, was kindly provided by D. Bramblett of Texas Tech University Health Sciences Center.

Cell counts were conducted on sections of an area of 250X250 μm^2 (H&E) or 230X230 μm^2 (immunofluorescence) near the central retina. For each group, at least three animals were analyzed. Two-tailed Student's t test was used to assess statistical significance.

Electroretinogram

Eight *Lhx4*–null mice and eight control mice at 2-month-old were used for ERG study. Mice were allowed to dark-adapted overnight. Under dim red light, mice were anesthetized with ketamine/xylazine. The pupils were dilated with Tropicamide, and mice were placed on a heating pad at 37°C to maintain body temperature. ERG was recorded using a Burian Allen electrode (0.3-1000 Hz; Hansen Ophthalmic Development Laboratory, Coralville, IA). Full-field ERGs were recorded following the International Society for Clinical Electrophysiology of Vision standard protocol. Single-flash cone response was recorded after 10 minutes of light adaption.

RNA-Sequencing (RNA-Seq)

For RNA-Seq experiment at each developmental stage (P6 and P7), three *Lhx4* null mice and three control mice were used, and the retinas of each mouse were collected as one RNA-Seq sample. Retinas were isolated by removing retinal pigment epithelium from eyecups under a dissecting microscope. Total RNA was extracted with RNeasy kits (QIAGEN, Valencia, CA) in accordance with the manufacturer's protocol. The cDNA libraries were generated by using TruSeq RNA Sample Prep Kit (Illumina) and were sequenced on the Illumina HiSeq X ten sequencer (Illumina). Sequence depths are from 19M to 40M reads per library and sequence parameters were set at 150 bp paired-end reads. Short reads mapping and differentially expressed gene identification were performed using Tophat (RRID: SCR_013035) and Cufflinks (RRID: SCR_014597) (Trapnell et al., 2012). Gene ontology enrichment was analyzed by using Enrichr (http://amp.pharm.mssm.edu/Enrichr/; Kuleshov et al., 2016). The accession number for the RNA-seq data from *Lhx4* null retinas and control reported in this paper is GEO: GSE126942 and GSE127771.

Co-immunoprecipitation and western blot

Wild-type retinas at P8 were collected and lysed in the RIPA buffer (ThermoFisher, #89900) with a protease inhibitor cocktail (Roche, #04693124001). Three biological replicates were performed. Cell lysate was centrifuged for 20 min at 12,000 rpm at 4°C. The supernatant was incubated with anti-ISL1 (Abcam Cat# ab20670, RRID:AB_881306) at 4°C overnight. Then, the protein A/G agarose beads (Santa Cruz Biotechnology Cat# sc-2003, RRID:AB_10201400) was added into the sample and incubated at 4°C for 4 hours. After four washes with RIPA buffer, the sample was suspended with 2x Laemmli Sample Buffer (Bio-Rad #1610737) and boiled for 10 min. Then, the sample was loaded into SDS-PAGE gel, and ran for 2 hours at 100 V. The proteins were transferred from the gel to PVDF membrane using Mini Trans-Blot electrophoretic transfer cell (Bio-Rad). Next, the membrane was blocked with the blocking buffer (5% milk in TBST) for 1 hour at RT, and incubated with anti-LHX4 (1:1000, Proteintech # 11183-1-AP) at 4°C overnight. Then, the membrane was washed three times with TBST for 5 min each, it was incubated with VeriBlot for IP Dectection Reagent (1: 1000, Abcam #ab131366) for 1 hour at RT. After the membrane was washed four times with TBST for 5 min each, detection was done with ImageQuant LAS4000 biomolecular imager (GE Healthcare).

Subretinal DNA electroporation

Electroporation was performed as previously described (de Melo and Blackshaw, 2018; Matsuda and Cepko, 2004). In brief, P0 mice were anesthetized by chilling on the ice. Eyes were opened by cutting the fused eyelid junction and then a small incision was made in the sclera near the lens using 30-guage needle. Approximately 0.5 μL of DNA solution (2.5-5 μg/μl) in PBS containing fast green as a tracer was injected into the subretinal space through the incision by using a Hamilton syringe with a 33-gauge blunt-ended needle, and squared pulses (80V; five pulses of 50 ms duration with 950 ms interval) were applied using ECM830 (BTX) with tweezer-type electrodes (model 520, BTX).

Droplet Digital PCR (ddPCR) quantification

Total RNA was separately extracted from three control and three *Lhx4* null mice at adult with RNeasy Mini Kits (QIAGEN, Valencia, CA) in accordance with the manufacturer's protocol. cDNA was synthesized from total RNA using iScript cDNA Synthesis Kit (Bio-Rad). ddPCR was performed by using QX200 Droplet Digital Systems (Bio-Rad). QX200 EvaGreen Digital PCR Supermix (Bio-Rad) was used for the ddPCR reaction. The analysis was performed on the QuantaSoft Analysis Pro (Bio-Rad). The housekeeping gene *Actb* was used for internal control.

QUANTIFICATION AND STATISTICAL ANALYSIS

Statistical analyses were conducted and graphs were constructed using Prism version 6.0. For each analysis, results from independent animals were treated as biological replicates ($n \geq 3$). The two-tailed Student's t test was performed to determine whether differences in cell numbers and ERGs were statistically significant. Data are represented as mean ± SD. Statistical significance was set at $p < 0.05$.

Cell Reports, Volume *32*

Supplemental Information

LIM-Homeodomain Transcription Factor LHX4

Is Required for the Differentiation of Retinal Rod

Bipolar Cells and OFF-Cone Bipolar Subtypes

Xuhui Dong, Hua Yang, Xiangtian Zhou, Xiaoling Xie, Dongliang Yu, Luming Guo, Mei Xu, Wenjun Zhang, Guoqing Liang, and Lin Gan

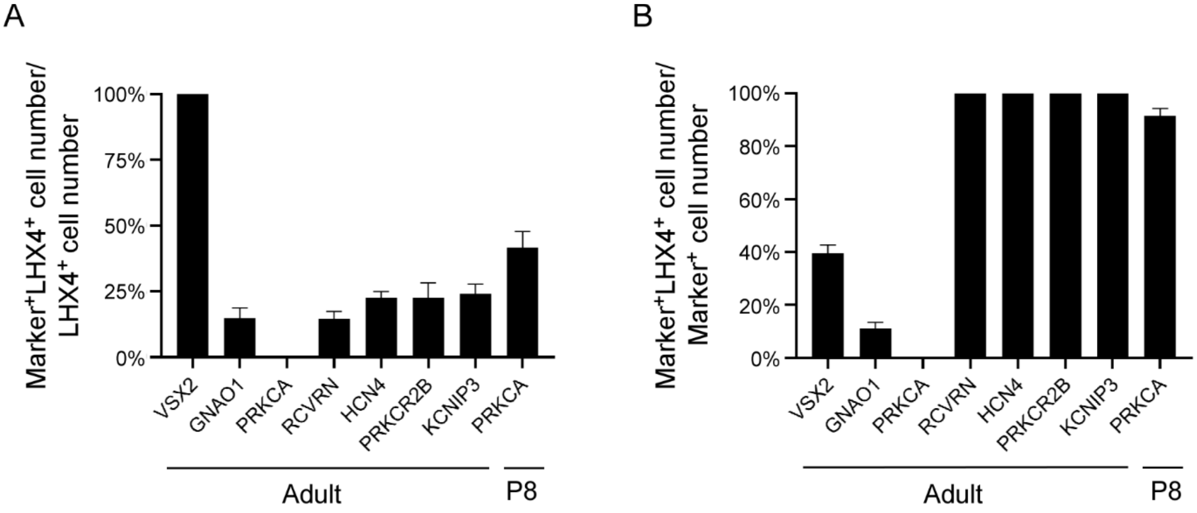

Figure S1. Quantification of LHX4 expression in bipolar subtypes. Related to Figure 1. (A) The percentage of LHX4^{+} cells expressed different bipolar subtype markers. (B) The percentage of different bipolar subtype markers expressed LHX4.

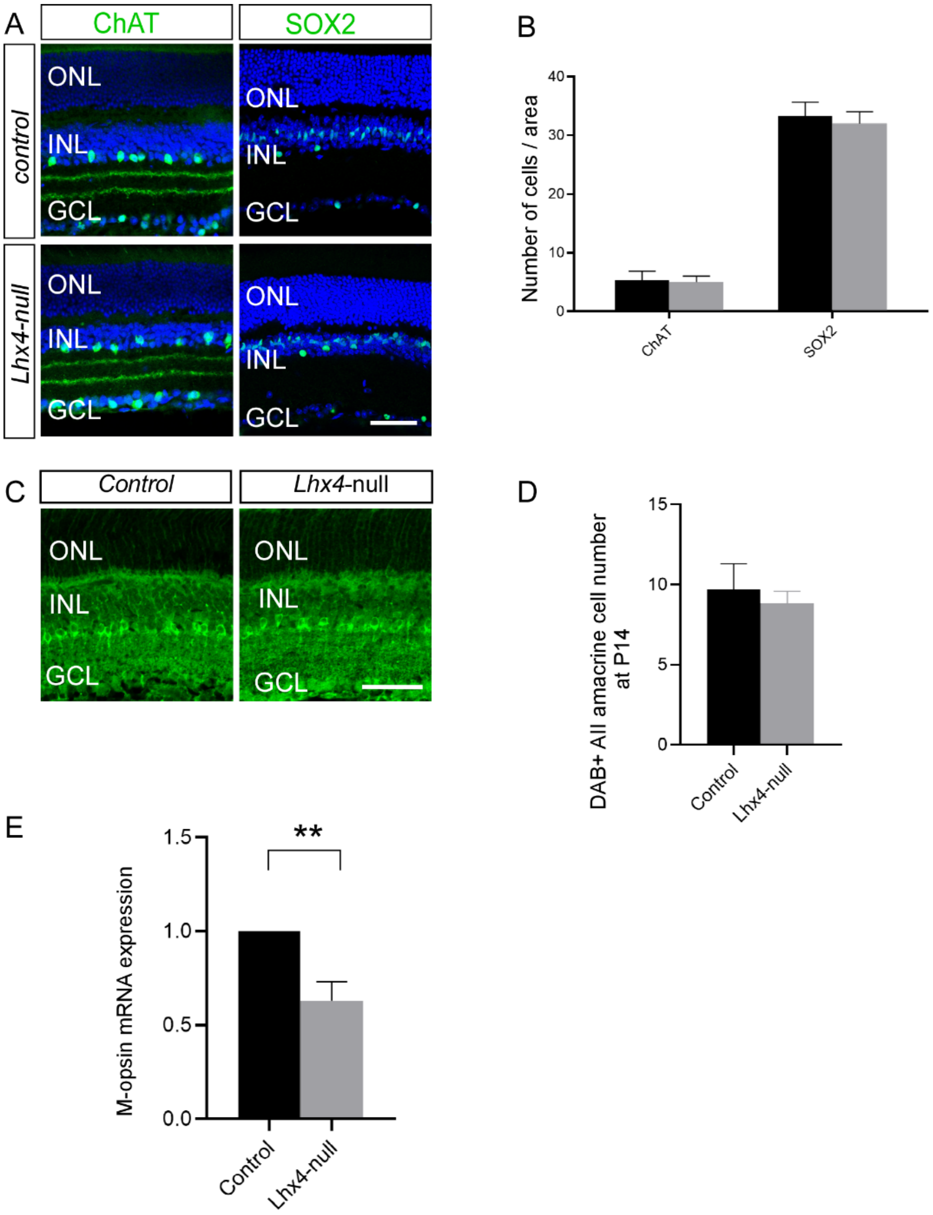

Figure S2. The effect of *Lhx4* inactivation on the generation of Müller glial cells and amacrine cells and the expression of M-opsin. Related to Figure 3. (A) The number of $SOX2^+$ Müller glial cells and $ChAT^+$ starburst ACs in the *Lhx4*-null retina was comparable to that in the control at adult. (B) Quantification of the cell numbers per imaging area (n≥3). (C) Immunolabeling for DAB1 showed that AII ACs were generated normally in the *Lhx4*-null retina at P14. (D) Quantification of $DAB1^+$ AII amacrine cell number in the *Lhx4*-null retina and control at P14. (E) ddPCR analysis showed *M-opsin* mRNA was significantly reduced by 37.0% in the adult *Lhx4*-null retina. Data are represented as mean ± SD. **$p < 0.01$. Scale bars, 50 μm.

A

Category	Gene
Glutamate receptor signaling pathway	*Grm6*, *Grik1*
Anterograde trans-synaptic signaling	*Gabrr3*, *Grik1*, *Mchr1*
Chemical synaptic transmission	*Gabrr3*, *Grik1*, *Mchr1*
Modulation of chemical synaptic transmission	*Grm6*, *Grik1*
Regulation of synaptic transmission, glutamatergic	*Grm6*, *Grik1*
Adenylate cyclase-inhibiting G-protein coupled receptor signaling pathway	*Grm6*, *Mchr1*
Indolalkylamine metabolic process	*Tph1*, *Aanat*
Cellular biogenic amine biosynthetic process	*Tph1*, *Aanat*
Ion transmembrane transport	*Gabrr3, Atp4a, Ryr3*
Regulation of cell communication	*Grik1, Samsn1*

B

Category	Gene
Glutamate receptor signaling pathway	*Trpm1*, *Grm6*, *Grik1*
Anterograde trans-synaptic signaling	*Gabrr3*; *Glra1*, *Gabrr1*, *Grik1*
Chemical synaptic transmission	*Gabrr3*; *Glra1*, *Gabrr1*, *Grik1, Cdh8*
Gamma-aminobutyric acid signaling pathway	*Gabrr3*; *Gabrr1*
Synaptic transmission, glutamatergic	*Grik1, Cdh8*
G-protein coupled glutamate receptor signaling pathway	*Trpm1, Grm6*
Calcium ion transport	*Trpm1*, *Trpc7*, *Ccr5*, *Ryr3*
Calcium ion transmembrane transport	*Trpm1*, *Trpc7*, *Ryr3*
Response to alcohol	*Glra1*, *Ccr5*
Ion transmembrane transport	*Gabrr3*; *Glra1*, *Gabrr1*, *Ryr3*

Figure S3. RNA-Seq analysis shows that loss of *Lhx4* results in differentially expressed genes associated with synapses. Related to Figure 5. (A) Genes downregulated by loss of *Lhx4* in the top 10 categories of GO enrichment at P6. (B) Genes downregulated by loss of *Lhx4* in the top 10 categories of GO enrichment at P7.

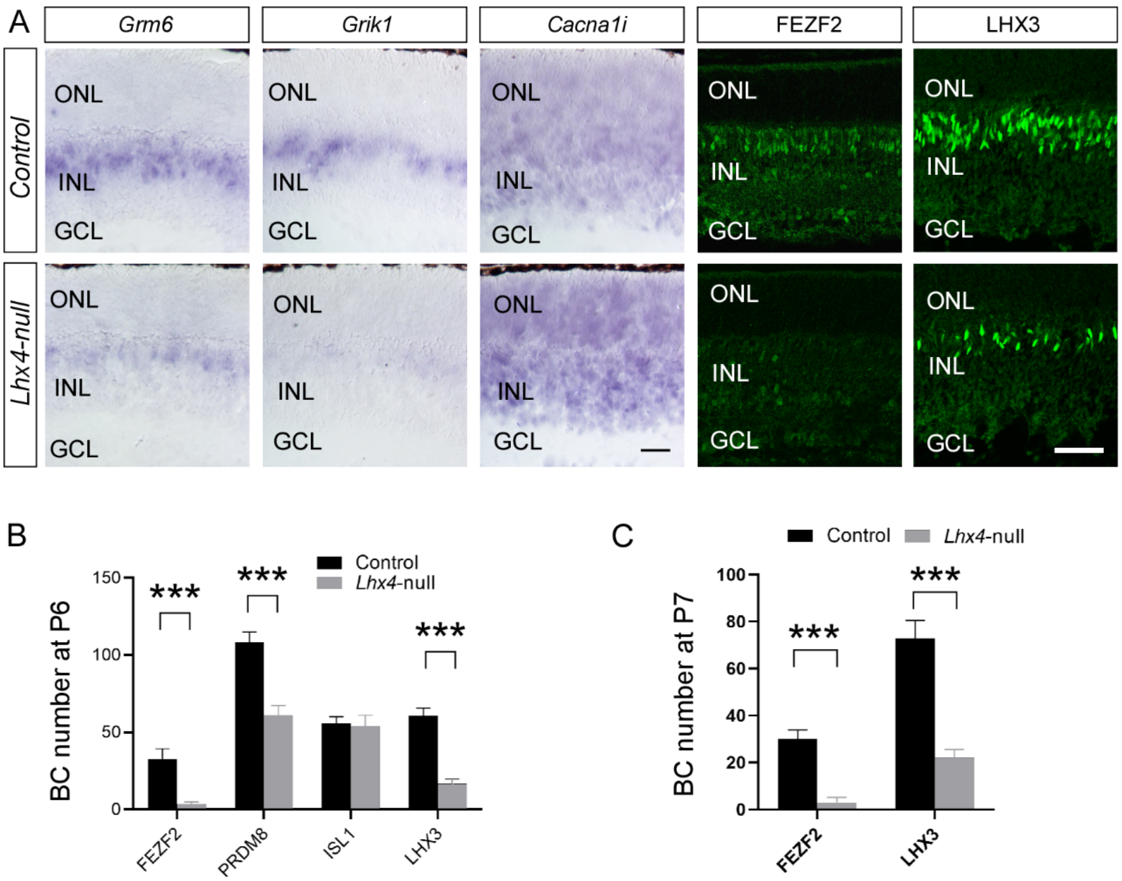

Figure S4. In situ hybridization and immunostaining confirmation of differentially expressed genes. Related to Figure 5. (A) The expression of *Grik1, Grm6*, LHX3, and FEZF2 was significantly reduced in *Lhx4*-null retinas, while the expression of *Cacna1i* was increased in *Lhx4*-null retinas at P7. (B) Quantification of FEZF2$^+$, PRDM8$^+$, ISL1$^+$, and LHX3$^+$ BCs in the *Lhx4*-null retina and control at P6. (C) Quantification of FEZF2$^+$ and LHX3$^+$ BCs in the *Lhx4*-null retina and control at P7. Data are represented as mean ± SD. ***p < 0.001. Scale bars, 50 µm.

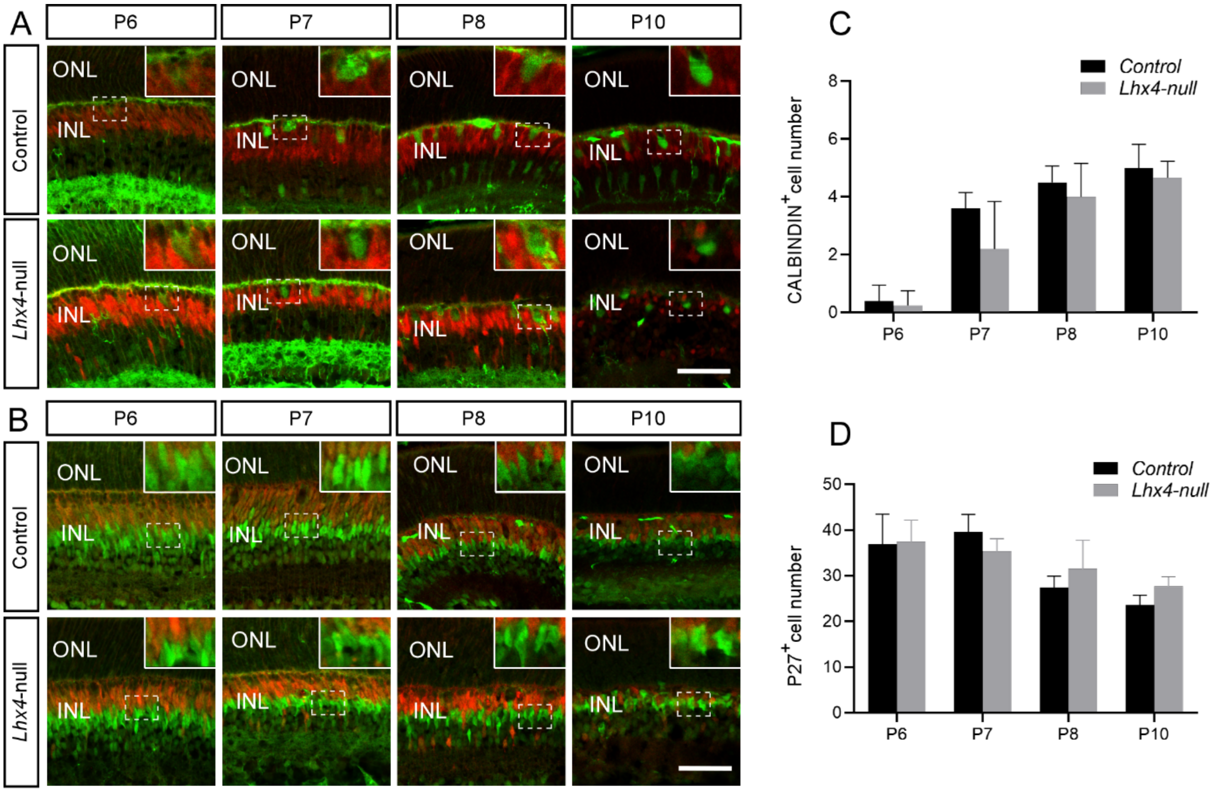

Figure S5. The expression of tdTomato is not detected in the horizontal cells and Müller glia cells in the *Lhx4*-null retina. Related to Figure 6. (A) CALBINDIN^{+} horizontal cells did not express tdTomato in the control or the *Lhx4*-null retina at P6, P7, P8 and P10. (B) No tdTomato expression was detected in Müller glia cells (p27kip1^{+}) of the control and the *Lhx4*-null retina at P6, P7, P8 and P10. (C) Quantification of CALBINDIN^{+} horizontal cell number in the *Lhx4*-null retina and control. (D) Quantification of P27^{+} Müller glia cell number in the *Lhx4*-null retina and control at P6, P7, P8 and P10. Scale bars, 50 µm.
